# Influence of Electrostatic Field on Optical Rotation of D-Glucose Solution: Experimental Research for Electric Field-Induced Biological Effect

**DOI:** 10.3390/molecules29204898

**Published:** 2024-10-16

**Authors:** Quanlin Guo, Dezhi Gou, Chenxi Zhao, Yun Ma, Chaojun Chen, Junxi Zhu

**Affiliations:** School of Electronic and Information Engineering, China West Normal University, Nanchong 637000, China; m18398999056@163.com (Q.G.); 15775649088@163.com (C.Z.); 17781805450@163.com (Y.M.); 15160321639@163.com (C.C.); 18284648157@163.com (J.Z.)

**Keywords:** electrostatic field, D-glucose solution, optical rotation, electric field-induced biological effect

## Abstract

At present, the effects of environmental electromagnetic irradiation on the metabolism of organisms have attracted extensive attention, but the mechanism is still not clear. D-glucose plays an important role in the metabolism of organisms. In this work, the change in the optical rotation of D-glucose solution under an electrostatic field is measured experimentally, so as to explain the mechanism of the electric field-induced biological effect. The experimental results show that the electrostatic field can alter the optical rotation of D-glucose solution at different temperatures. Under the different strengths of electrostatic field, the specific rotation of D-glucose solution increases at different temperatures; the maximum increase can reach 2.07%, but the effect of temperature and electric field strength on the rotation increment is nonlinear and very complex. Further, it turns out that the proportion of α-D-glucose in solution increases by up to 3.25% under the electrostatic field, while the proportion of β-D-glucose decreases by as much as 1.75%. The experimental study confirms that electrostatic field can change the proportion of two conformation molecules (α and β-D-glucose) in D-glucose solution, which can provide a novel explanation for the mechanism of the electric field-induced biological effect.

## 1. Introduction

Electromagnetic irradiation is widely used in the field of wireless communication, which dramatically increases the artificial electromagnetic radiation level on the earth [1,2,3]. Whether environmental electromagnetic radiation affects human life and health and electromagnetic biological effects have once again attracted people’s attention [4,5,6,7,8]. Shi et al. observed the effect of microwave irradiation on sleep of mice [9], and long-term electromagnetic radiation was verified a potential factor affecting sleep. Shneider et al. studied the effect of microwave radiation on myelin nerve fibers [10], and the results showed that microwave radiation changed the threshold value of the action potential in the axons of the neural network. Yarovsky et al. researched the effect of microwave radiation on the molecular structure of poly-peptides [11], and microwave radiation could induce the production of poly-peptides with different conformations. These studies show that electromagnetic radiation does affect organisms, cells, and molecules, but the mechanism of electromagnetic biological effects remains unclear. It is agreed that electromagnetic irradiation can have an important effect on the metabolism of organisms through thermal effects, but the influence of non-thermal effects is still controversial. This work will attempt to study the electrostatic field, so as to exclude the role of thermal effect and prove the existence of electromagnetic non-thermal effect.

Chiral molecules have optical rotation and electric dipole moments, and are polarized under electromagnetic irradiation, which leads to changes in the conformation of chiral molecules [12,13,14,15,16]. Schnell et al. proposed a new method of a pulsed microwave control of the enantiomeric transformation of chiral molecules in gas phase [17]. The enantiomeric selectivity of chiral molecules is controlled at the molecular level by using a three-way resonant pulsed microwave nonlinear coherence technique. Swager et al. found the induction of electric field chirality in columnar liquid crystals [18]. Taking a tetraphenyl disk-like molecule as the object, it is found that the enantiomeric hexagonal columnar liquid crystals transformed into helical columnar structures under the action of an electric field, which proved the induction of electric field chirality. Zou et al. studied the asymmetric topological polymerization of non-chiral diethyl monomers induced by a chiral electromagnetic field [19]. Yamada et al. measured the change in the excess enantiomer percentage in the Nazarov cyclization reaction under microwave irradiation [20,21], and proved that microwave has isomerization transformation and microwave non-thermal effects. These experiments show that the electromagnetic field can indeed affect chiral molecules, but it does not rule out the role of thermal effect. This work will use electrostatic field to act on chiral molecules, and observe experimentally the change in the optical rotation of the chiral molecules under the electrostatic field.

Glucose is one of the most widely available sugars and plays an important role in biochemistry [22], not only as a core molecule of metabolism, but also as a participant in a variety of physiological and biochemical processes. The effect of electromagnetic field on glucose has been reported [23]. Pagnotta et al. researched the effect of microwave radiation on the optical rotations of glucose in an ethanol aqueous solution [24], and it was found that the contents of α-D-glucose and β-D-glucose in the solution were changed by microwave heating compared with the traditional water bath heating at the same temperature. Fan et al. simulated the effect of 2.45 GHz microwave radiation on molecules in a glucose aqueous solution [25]. Jian et al. studied the effects of pulsed electric field (PEF) on the physicochemical properties of bovine serum albumin–glucose and bovine serum albumin–mannose couplings. The results showed that PEF not only promoted the Maillard reaction of bovine serum albumin with glucose or mannose, but also alleviated the adverse reaction of browning [26]. This work is to study the change in the optical rotation of glucose solution under electrostatic field.

In this work, first, the experimental system was set up to measure the optical rotation of D-glucose solution under the electrostatic field. The temperatures were 5, 10, 16, 20, 25, and 30 °C, and the electric field strengths were 31.25, 62.5, 125, 187.5, and 250 V/m. Then, the changes in the two conformations of glucose (α and β-D-glucose) in the solution were further calculated. Finally, the mechanism for the effect of electrostatic field on optical rotation was analyzed, which provided new insights for the mechanistic explanation of electric field-induced biological effect.

## 2. Results and Discussion

### 2.1. Influence of the Temperature on the Optical Rotation of D-Glucose 

**Figure 1:**
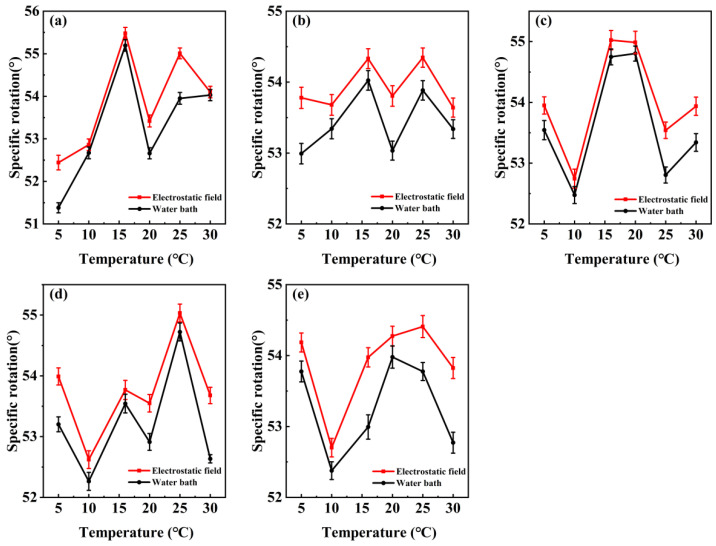
Specific rotation of D-glucose solution with the different temperatures under an electrostatic field; (**a**–**e**) represent the five different strength electrostatic field. (**a**) 31.25 V/m. (**b**) 62.5 V/m. (**c**) 125 V/m. (**d**) 187.5 V/m. (**e**) 250 V/m. The red line shows the electrostatic field, and the black line shows the water bath.

The variation in the specific rotation of D-glucose solution with temperature (Figure 1) was measured at different temperatures. The specific rotations of D-glucose solution fluctuate as temperature increases from 5 °C to 30 °C. The measurements of specific rotations of D-glucose solution at different temperatures and electrostatic fields (Table 1) were shown. Through the standard deviation of 15 measurements of specific rotations, it can be found that most of the standard deviation under the electrostatic field is smaller than that without the electric field. This indicates that the temperature will produce certain fluctuations in the specific rotations in the absence of electrostatic field. Kholmanskiy observed that the abnormal fluctuations in the specific rotation of D-glucose solution at temperatures below 15 °C and the restructuring of water clusters can affect the chirality of sugars within the temperature range of 20 to 25 °C [27]. This shows that the specific rotation of D-glucose solution has a certain fluctuation, which is related to temperature. At 31.25, 125, and 187.5 V/m (Figure 1a,c,d), there are obvious peaks in the curve of specific rotation. At 62.5 and 250 V/m (Figure 1b,d), the specific rotation fluctuates relatively smoothly. The electrostatic field generally shows slightly higher specific rotation compared to the water bath, and the maximum increase rate of the specific rotation can reach 2.07%. The fluctuation in specific rotation caused by temperature indicates that the molecules may undergo conformation changes at different temperatures.

**Figure 2:**
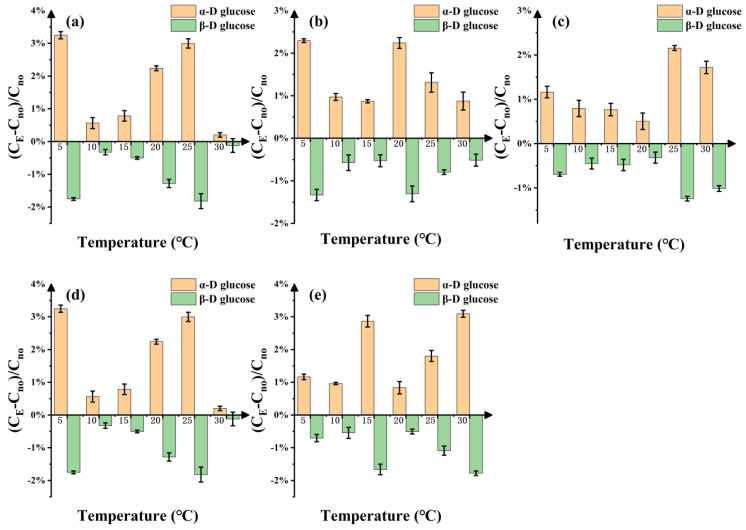
Variation in α-D-glucose and β-D-glucose in D-glucose solution with the different temperatures under electrostatic field; (**a**–**e**) represent the five different strength electrostatic fields. (**a**) 31.25 V/m. (**b**) 62.5 V/m. (**c**) 125 V/m. (**d**) 187.5 V/m. (**e**) 250 V/m. The yellow column shows the relative change rate of the proportion of α-D-glucose under the electrostatic field with respect to the water bath. The green column shows the relative change rate of the proportion of β-D-glucose.

According to the specific rotation of D-glucose solution, the proportion of two conformation components (α and β-D-glucose) in D-glucose solution was further analyzed, and the relative change rate of these two conformation components relative to the water bath under the electrostatic field (Figure 2) was obtained. Overall, the relative change rate of α-D-glucose is all positive and the amplitude gradually increases with increasing temperature. In contrast, the relative change rate of β-D-glucose is all negative and the amplitude also increases with temperature. The positive change in α-D-glucose and the negative change in β-D-glucose basically show a symmetric or mirror relationship. At a specific temperature of 30 °C with the electric fields of 31.25 and 187.5 V/m (Figure 2a,d), the relative change rates of α-D-glucose and β-D-glucose are relatively little. These regularities emphasize that the effects of temperature on D-glucose are very complex and may be related to their chemical or biological properties.

### 2.2. Influence of the Electrostatic Field on the Optical Rotation of D-Glucose

**Figure 3:**
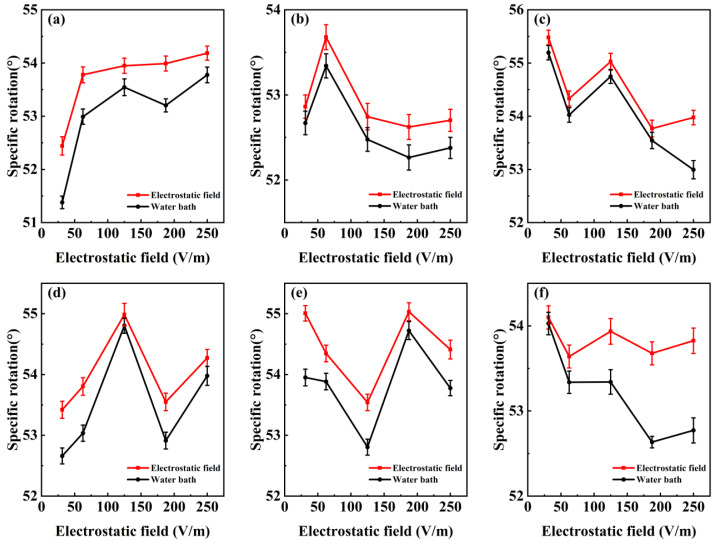
Specific rotation of D-glucose solution under the different electrostatic fields: (**a**–**f**) represent the six different temperatures. (**a**) 5 °C. (**b**) 10 °C. (**c**) 16 °C. (**d**) 20 °C. (**e**) 25 °C. (**f**) 30 °C. The red line shows the electrostatic field, and the black line shows the water bath.

The variation in the specific rotation of D-glucose solution with electrostatic field (Figure 3) was measured under different electrostatic fields. The specific rotation of D-glucose solution under the electrostatic field is higher than that under the water bath, and with the increase in electric strength, the specific rotation shows the obvious trend of fluctuation. The specific rotation gradually increases when the electric strength increases from 5 V/m to 100–150 V/m, and reaches the peak value. Subsequently, when the electric strength continues to increase to 200 or 250 V/m, the specific rotation decreases. The specific rotation under the electrostatic field has an obvious peak value with the specific rotation, while the specific rotation with the water bath changes more gently. This is possibly because the electrostatic field affects the orientation or polarization of molecules, thereby altering their optical activity.

**Figure 4:**
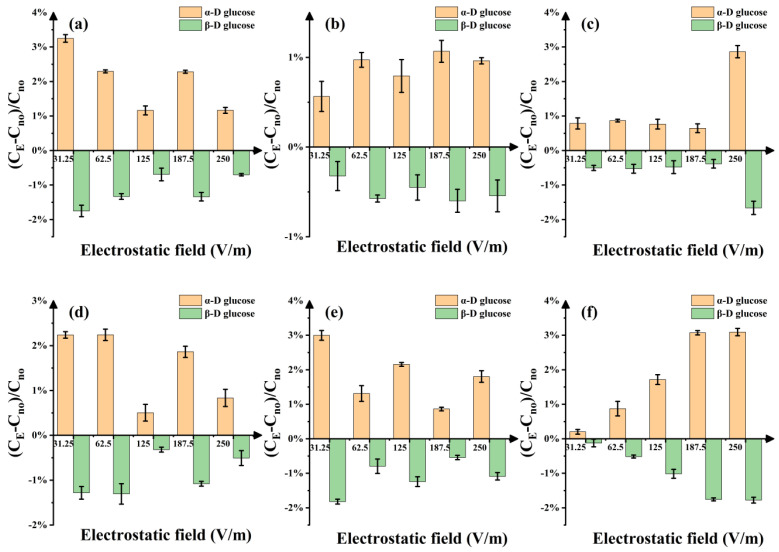
Variation in α-D-glucose and β-D-glucose in D-glucose solution under different electrostatic fields: (**a**–**f**) represent the six different temperatures. (**a**) 5 °C. (**b**) 10 °C. (**c**) 16 °C. (**d**) 20 °C. (**e**) 25 °C. (**f**) 30 °C. The yellow column shows the relative change rate of the proportion of α-D-glucose under the electrostatic field with respect to the water bath. The green column shows the relative change rate of the proportion of β-D-glucose.

The relative change rate of the proportion of two conformation components (α and β-D-glucose) in D-glucose solution under the electrostatic fields compared to the water bath (Figure 4) was obtained. As the electric strength increased, the positive relative change rate of α-D-glucose generally showed an increasing trend by up to 3.25%. The negative change in β-D-glucose is deepened by as much as 1.75%, and this trend is especially obvious at higher electrostatic field intensity (such as 250 V/m). At 10 °C (Figure 4b), the two conformation components change smoothly with the electric strength, while at other temperatures, the relative change rate is more fluctuant and there will be some obvious peaks, especially at 10 °C and 30 °C (Figure 4c,f). These results show that the electrostatic field has a very complex effect on D-glucose solution, and the changes in the proportion of the two conformation components also indicate that the electrostatic field may cause changes in the biochemical processes in D-glucose solution.

### 2.3. Discussion on the Mechanism of the Effect of the Electrostatic Field on the Optical Rotation of D-Glucose Solution

**Figure 5:**
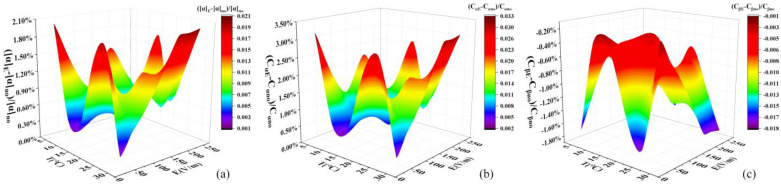
Three-dimensional surface diagram on the relative change rates of specific rotation, α-D-glucose, and β-D-glucose with temperature and electric field strength. (**a**) Specific rotation. (**b**) Proportion of α-D-glucose. (**c**) Proportion of β-D-glucose.

**Figure 6:**
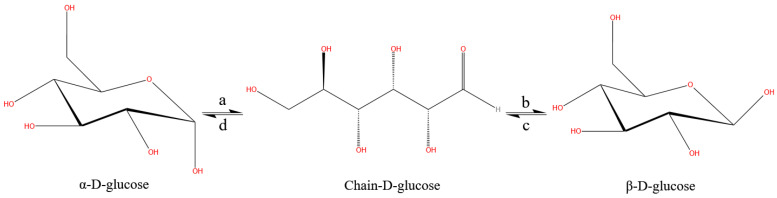
Multiple chemical reactions in D-glucose solution. **a**, **b**, **c**, and **d** represent the chemical reactions between these three molecular structures, respectively.

From the relative change rates of specific rotation and the proportion of α-D-glucose and β-D-glucose (Figure 5), the specific rotation (Figure 5a) increases under the electrostatic field, the proportion of α-D-glucose (Figure 5b) increases and the proportion of β-D-glucose (Figure 5c) decreases. In the D-glucose solution, there is a mutarotation phenomenon, that is, the two conformations α-D-glucose and β-D-glucose pass through the chain structure of D-glucose to reach chemical equilibrium. The mutarotation phenomenon of D-glucose solution involves multiple reaction processes and is a reversible reaction system (Figure 6). Based on the increase in α-D-glucose and the decrease in β-D-glucose in solution, it can be thought that the enhancement of reaction d shifts the reaction equilibrium toward α-D-glucose.

**Figure 7:**
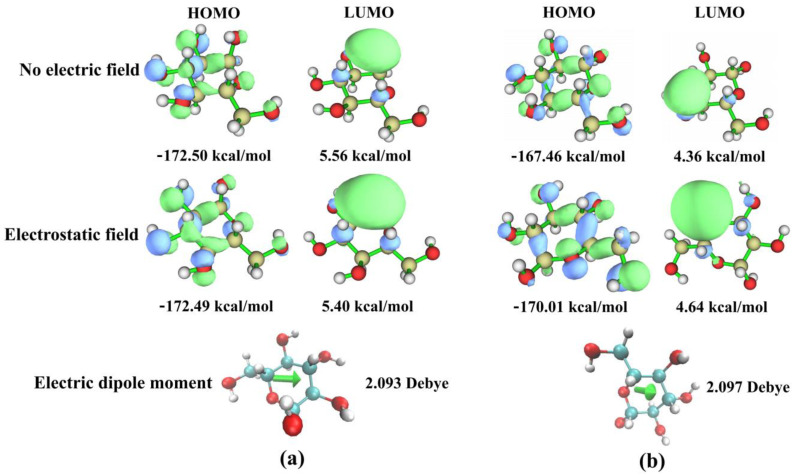
The HOMO, LUMO, and electric dipole moment of the α-D-glucose (**a**) and β-D-glucose (**b**). The top four diagrams show no electric field, the middle row four diagrams show electrostatic field, and the bottom two diagrams show electric dipole moments.

In order to understand the mechanism of the electrostatic field’s effect on the optical rotation of D-glucose solution, the highest occupied molecular orbital (HOMO), the lowest occupied molecular orbital (LUMO), and the electric dipole moments of α-D-glucose and β-D-glucose molecules (Figure 7) were calculated by quantum chemistry. The energy gap between the LUMO and HOMO of the α-D-glucose is 178.06 kcal/mol without the electric field, but 177.89 kcal/mol with the electrostatic field. In the same way, the energy gap of the β-D-glucose is 171.82 kcal/mol without the electric field, but 174.65 kcal/mol with the electrostatic field. The energy gap can represent the stability of the molecule, and it is found that α-D-glucose is more stable than β-D-glucose without and with the electrostatic field. To a certain extent, it can be explained that α-D-glucose is the majority in D-glucose solution.

Clearly, there may be more to this phenomenon than the above. Magazù et al. revealed that the low intensity electromagnetic field can affect the secondary structure of proteins, due to the vibration and bending of certain chemical bonds within the molecule affected by electromagnetic fields [28,29]. In D-glucose solution, the electrostatic field can affect the bending and vibration of the internal chemical bonds of molecules, resulting in the change in optical rotation characteristics. In addition, Magazù et al. studied the behavior of water in the montmorillonite–water mixture and the dynamic changes in its hydrogen bond network [30]. Hydrogen bonds also play a very important role in D-glucose solution, and the electrostatic field should also affect the optical rotation of D-glucose by affecting the hydrogen bond network between water and D-glucose. Unfortunately, the understanding of complex chemical processes in the D-glucose solution under the electrostatic field is still unclear. Subsequent work will study the kinetic mechanism of the effect of the electrostatic field on the optical rotation of D-glucose solution through molecular dynamics simulation and quantum chemistry methods.

## 3. Experiment and Method

### 3.1. Experimental System

**Figure 8:**
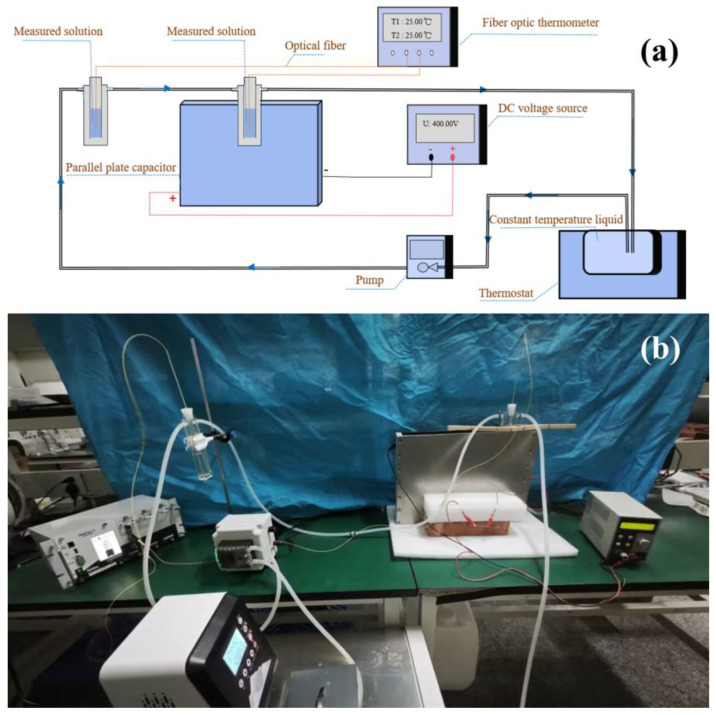
Diagram of experimental system. (**a**) Schematic diagram. (**b**) Actual experimental system.

The experimental system under the electrostatic field (Figure 8) was designed as follows. The system had a parallel plate capacitor connected at both ends of the DC voltage source to generate an electrostatic field in the capacitor. Two double casing tubes were also used to hold the tested solution, which was placed in the casing, and the constant temperature liquid was circulated through the pump outside the casing. The two tubes were placed inside and outside parallel plate capacitors, respectively, for electrostatic field and water bath experiments. A thermostat was used in the experiment, and the constant temperature liquid in the thermostat was circulated to the outside casing through the water pump to ensure the same temperature of the measured solution in the inner casing. The temperature test used a fiber optic thermometer, and the temperature of the solutions under the two conditions were almost the same, and the error range was plus or minus 0.1 °C.

### 3.2. Experimental Materials

The D-glucose concentration used in the experiment was 0.5011 g/100 mL, and the D-glucose purity was 99.7%. This required 0.5011 g of D-glucose and 100 mL of deionized water. The D-glucose solution was prepared and left for 24 h before using.

### 3.3. Measurement of Optical Rotation

The experimental variables were the six temperatures of 5, 10, 16, 20, 25, and 30 °C and the five electric field strengths of 31.25, 62.5, 125, 187.5, and 250 V/m. At the same temperature, the water bath and the electrostatic field were applied to the measured liquid (D-glucose solution) for 30 min. In the same batch of experiments, both microwavable and non-microwavable samples used the same batch configuration solution, and the two samples were not reused after the end of the experiment. Then, the specific rotation of the D-glucose solution treated by the two methods was measured by the SGW-531 fully automatic optical rotation tester (INESA Physico-optical Instrument Co., Ltd., Shanghai, China). The test solution was placed in the test tube with a length of 100 mm, and then the test tube was placed in the tester for testing. All test data including the optical rotation and specific rotation of D-glucose solution, their average values, and their standard deviations are given (Supplementary Materials).

### 3.4. Test Data Analysis

The intrinsic specific rotation of α-D-glucose [α]_α_ was +112°, the intrinsic specific rotation of β-D-glucose [β]_β_ was +18.7°, and the [α] was the specific rotation of the tested D-glucose. In the D-glucose solution, the proportion of α-D-glucose and β-D-glucose and the specific rotation satisfy the relationship [α] = C_α_[α]_α_ + C_β_[β]_β_ and C_α_ + C_β_ = 1, C_α_ and C_β_ are the proportions of α-D-glucose and β-D-glucose in the D-glucose solution. According to this relationship, the proportion of the two components can be obtained. Finally, the relative change rate of the specific rotation of the D-glucose solution and the proportion of the α-D-glucose and β-D-glucose under the electrostatic field with respect to the water bath is obtained.

### 3.5. Quantum Chemical Computations

To explain the mechanism for the electrostatic field’s effect on the optical rotation of D-glucose solution, we need to analyze the molecular stability the α-D-glucose and β-D-glucose molecules. We employed the ab initio quantum chemical calculation to optimize the structure of α-D-glucose and β-D-glucose molecules using the Gaussian 03 program package [31]. Density functional theory calculations were performed for optimization using the B3LYP [32,33] approach with the 6-311+G (d) basis set [34]. The electric dipole moment was derived from the molecular orbitals using the Multiwfn 3.3.8 program package [35] to gain further the electric dipole moment vector. Then, by analyzing the molecular orbitals, the highest occupied molecular orbital (HOMO) and the lowest occupied molecular orbital (LUMO) were obtained, and the energy gap between the LUMO and HOMO was calculated. In addition, the electric field of 250 V/m along the direction of the electric dipole moment vector was added in the ab initio quantum chemical calculation, and the effect of the electric field on the energy gap between the LUMO and HOMO was analyzed.

## 4. Conclusions

In this paper, the influence of the electrostatic field on the optical rotation of D-glucose solution is studied. The experimental results show that the electrostatic field can alter the specific rotation of D-glucose solution and the proportion of the α-D-glucose and β-D-glucose at different temperatures. Under the different strengths of electrostatic fields, the specific rotation of D-glucose solution increases at different temperatures, the maximum increase rate can reach 2.07%, but the effect of temperature and electric field strength is nonlinear and very complex. Meanwhile, the proportion of α-D-glucose in solution increases by up to 3.25% under the electrostatic field, while the proportion of β-D-glucose decreases by as much as 1.75%. In addition, the effect of the electrostatic field on the optical rotation of D-glucose solution is probably related to the effect of electric field on the intramolecular chemical bond, the hydrogen bond between water and D-glucose and the molecular stability of α-D-glucose and β-D-glucose. The effect of electromagnetic field on bioactive molecules such as glucose is directly related to the metabolism of organisms and require continuous in-depth research. This work focuses on the influence of electrostatic field on the optical rotation of D-glucose, which is helpful for understanding and studying the electric field-induced biological effect.

## Figures and Tables

**Table 1:** The measurements, averages, and standard deviations of the specific rotation of D-glucose solution at different temperatures and electrostatic fields.

T (°C)	E (V/m)	1	2	3	4	5	6	7	8	9	10	11	12	13	14	15	Average	Standard Deviation
5	0	51.30	50.24	50.47	51.31	51.22	51.83	52.00	50.78	50.91	50.88	52.03	51.93	51.67	52.11	52.01	51.38	0.62
	31.25	52.84	52.57	52.56	52.34	52.24	52.44	52.51	52.19	52.23	52.37	52.38	52.57	52.58	52.47	52.33	52.44	0.17
	0	54.46	54.18	54.35	53.69	53.58	52.19	51.85	52.46	52.62	52.50	52.51	52.82	52.33	52.55	52.78	52.99	0.84
	62.5	54.48	55.49	54.51	54.17	55.06	53.07	53.32	53.17	53.25	52.73	53.54	53.43	53.39	53.41	53.67	53.78	0.79
	0	53.04	52.10	52.13	53.02	52.91	54.92	54.39	54.57	54.36	54.52	53.24	53.37	53.27	53.81	53.52	53.54	0.87
	125	53.68	54.16	53.69	53.39	53.08	54.58	53.65	54.44	54.59	53.85	53.93	53.81	54.02	54.12	54.23	53.95	0.43
	0	54.74	54.84	54.87	54.74	54.82	53.44	52.13	52.07	53.06	52.30	52.02	52.34	52.30	52.15	52.24	53.20	1.19
	187.5	55.02	55.14	55.24	54.83	55.00	53.68	53.56	53.81	53.52	53.75	53.13	53.41	52.94	53.44	53.39	53.99	0.78
	0	54.84	54.85	54.77	54.91	54.76	53.24	53.34	53.15	53.20	53.12	53.14	53.32	53.27	53.43	53.32	53.78	0.75
	250	55.06	54.44	54.29	54.35	54.53	53.94	54.00	54.14	54.21	53.87	53.85	54.31	54.02	53.94	53.84	54.19	0.32
10	0	53.45	53.56	53.62	53.16	53.34	52.21	51.95	52.46	52.39	52.56	52.41	52.15	52.22	52.27	52.31	52.67	0.58
	31.25	52.50	52.06	52.57	52.87	52.42	53.29	53.15	52.91	53.00	52.51	53.31	52.98	53.01	53.22	53.14	52.86	0.37
	0	53.30	52.74	52.84	52.96	52.91	54.18	53.86	53.68	53.77	53.67	53.15	53.26	53.12	53.36	53.33	53.34	0.41
	62.5	52.58	52.18	52.31	51.98	52.45	54.82	54.88	54.46	54.41	54.86	54.21	54.19	53.96	53.88	54.01	53.68	1.06
	0	52.41	52.55	52.38	52.58	52.48	52.99	52.75	52.87	52.86	52.73	52.15	52.12	52.01	52.13	52.16	52.48	0.32
	125	52.35	53.14	52.54	52.94	52.74	53.61	53.24	53.10	53.06	53.23	52.44	52.02	52.36	52.27	52.12	52.74	0.48
	0	54.33	54.08	54.36	54.12	54.02	50.52	50.50	50.48	50.51	50.35	52.26	51.99	52.18	52.01	52.27	52.27	1.58
	187.5	54.00	54.00	53.96	54.34	54.13	51.52	51.49	51.70	51.10	51.19	52.53	52.21	52.44	52.57	52.15	52.62	1.16
	0	52.18	52.58	52.22	52.53	52.38	52.94	52.57	52.19	52.30	52.56	52.13	52.36	52.34	52.27	52.12	52.38	0.22
	250	52.50	52.90	52.65	52.75	52.70	53.37	52.77	52.45	52.82	52.64	52.90	52.64	52.67	52.23	52.54	52.70	0.25
16	0	55.07	54.99	55.10	55.10	55.17	55.31	55.30	55.28	55.19	55.76	56.19	55.31	55.86	54.05	53.99	55.18	0.57
	31.25	55.28	55.08	55.22	55.15	55.33	55.68	55.88	55.74	55.48	55.99	56.11	56.17	55.82	54.01	54.78	55.45	0.57
	0	54.12	54.09	53.58	53.83	53.98	54.09	53.89	53.57	53.69	53.67	55.69	55.63	55.33	55.40	53.67	54.28	0.79
	62.5	54.72	55.29	54.74	54.50	55.21	53.80	53.73	53.97	53.96	53.92	55.24	55.50	54.62	54.79	54.03	54.53	0.60
	0	54.70	54.70	54.76	54.54	54.23	54.32	54.56	55.08	54.79	54.90	54.73	54.96	55.18	55.26	54.61	54.76	0.29
	125	54.90	54.96	54.80	55.00	55.01	55.01	54.86	55.16	55.02	55.15	55.09	55.25	55.05	55.03	55.04	55.02	0.12
	0	53.50	53.42	53.29	53.47	53.49	53.93	54.02	54.24	54.06	53.86	53.78	52.61	53.80	52.26	53.42	53.54	0.53
	187.5	52.39	53.15	53.40	53.95	53.26	54.24	54.02	53.63	54.16	54.19	54.62	53.65	54.39	53.93	53.56	53.77	0.57
	0	53.80	53.15	53.56	53.30	53.12	53.16	53.12	52.76	52.93	52.56	53.23	52.97	53.17	53.25	52.80	53.12	0.31
	250	53.25	53.26	53.28	53.35	53.14	54.94	55.11	55.13	54.82	55.40	54.44	54.49	54.27	54.23	53.66	54.18	0.80
20	0	51.43	51.25	51.19	50.97	51.12	51.63	51.41	51.47	51.47	51.53	54.94	54.88	54.94	54.89	53.35	52.43	1.64
	31.25	52.61	52.36	52.39	52.28	52.28	52.61	52.04	52.05	52.30	51.90	55.83	55.78	55.49	55.24	53.87	53.27	1.52
	0	53.05	52.45	52.40	52.74	52.75	53.84	53.80	53.78	53.77	53.45	52.46	52.71	52.69	52.75	52.87	53.03	0.54
	62.5	53.15	53.07	53.19	53.00	53.15	54.95	54.96	55.08	55.26	55.18	53.32	53.40	53.16	53.32	52.90	53.80	0.95
	0	54.50	55.10	54.61	54.99	54.80	54.16	54.94	54.94	55.04	54.88	54.74	54.86	54.89	54.77	54.81	54.80	0.24
	125	54.61	55.36	54.70	55.26	54.98	54.75	55.32	55.05	54.75	55.06	55.03	54.96	55.15	55.01	54.77	54.99	0.23
	0	54.26	54.30	53.96	53.96	54.07	52.32	52.40	52.32	52.48	52.32	52.29	51.99	52.25	52.40	52.34	52.91	0.89
	187.5	53.98	54.24	54.01	54.21	54.11	53.19	53.14	53.40	53.23	52.94	53.44	53.35	53.26	53.24	53.50	53.55	0.43
	0	53.76	54.08	53.88	54.04	54.04	53.93	53.62	54.29	54.11	53.93	53.92	53.98	53.99	54.11	54.01	53.98	0.16
	250	53.57	53.73	53.93	53.84	53.97	54.28	54.65	54.59	54.53	54.89	54.04	54.55	54.57	54.34	54.62	54.27	0.40
25	0	54.90	54.50	54.38	54.45	54.45	53.64	53.47	53.44	53.38	53.76	53.94	53.58	53.99	53.59	53.79	53.95	0.47
	31.25	55.05	55.19	55.32	54.38	54.80	55.00	54.95	54.94	55.19	54.53	55.11	55.14	55.38	55.01	55.12	55.01	0.27
	0	53.64	53.84	54.04	53.06	53.56	52.31	51.21	51.53	51.75	51.57	56.44	56.85	55.88	56.27	56.24	53.88	2.01
	62.5	54.15	54.27	54.55	54.28	54.66	52.93	52.99	52.71	53.17	52.49	56.01	55.65	55.89	55.72	55.73	54.35	1.26
	0	52.71	52.76	52.64	52.98	52.81	52.96	52.86	52.83	52.94	52.60	52.88	52.95	52.77	52.83	52.56	52.81	0.13
	125	53.28	54.26	53.05	53.18	53.14	53.51	53.86	53.37	53.50	53.18	53.86	53.49	53.76	53.90	53.78	53.54	0.35
	0	52.74	52.62	52.44	52.53	52.56	55.87	55.77	55.74	55.90	55.68	55.98	55.39	55.76	55.86	55.99	54.72	1.57
	187.5	52.53	52.72	52.44	52.43	52.27	56.66	56.50	56.03	56.36	56.40	56.34	56.17	56.22	56.03	56.40	55.03	1.88
	0	53.50	53.23	53.32	53.70	53.62	53.99	53.72	54.11	54.12	53.85	53.98	53.88	54.01	53.86	53.77	53.78	0.27
	250	54.81	55.89	53.86	55.39	54.05	54.33	54.21	54.10	54.22	54.20	54.32	54.21	54.09	54.37	54.12	54.41	0.55
30	0	53.83	54.18	53.93	54.04	54.03	54.15	54.01	53.96	53.85	54.21	53.89	54.02	54.17	54.12	54.02	54.03	0.12
	31.25	54.00	54.20	53.96	54.15	54.10	53.76	53.48	54.42	53.79	54.68	54.00	54.15	54.12	54.32	54.29	54.09	0.29
	0	53.94	53.69	53.44	53.82	53.60	53.46	53.22	53.18	53.21	52.94	53.01	53.22	53.21	53.12	52.99	53.34	0.31
	62.5	53.65	53.84	54.41	53.56	54.24	53.16	53.54	53.13	53.40	53.36	53.55	53.53	53.85	53.78	53.60	53.64	0.35
	0	53.90	54.27	53.88	53.66	53.72	53.43	53.17	52.96	53.20	53.22	52.96	52.84	52.93	52.89	53.08	53.34	0.44
	125	55.02	55.16	54.72	54.82	55.42	54.42	53.86	53.82	54.12	54.10	52.75	52.50	53.07	52.56	52.71	53.94	1.01
	0	53.59	53.82	53.66	53.50	53.32	50.31	51.51	51.58	51.45	51.55	53.11	52.84	53.21	52.96	53.11	52.64	1.07
	187.5	54.13	53.69	53.48	53.67	53.38	52.72	53.80	53.68	54.13	53.92	53.59	53.44	53.87	54.01	53.65	53.68	0.35
	0	52.72	52.47	52.32	52.38	52.32	53.55	53.07	53.71	53.12	54.01	52.40	52.39	52.45	52.44	52.23	52.77	0.58
	250	53.11	54.06	54.43	54.38	53.17	53.94	54.74	53.79	54.18	53.49	53.60	54.14	53.59	53.34	53.41	53.83	0.49

## Data Availability

The original contributions presented in the study are included in the article; further inquiries can be directed to the corresponding author.

## Supplementary Materials

The following supporting information can be downloaded at: https://www.mdpi.com/article/10.3390/molecules29204898/s1, Tables S1–S6: Optical rotation and specific rotation of D-glucose solution, average values, and standard deviations; Table S1: The temperature is 5 °C; Table S2: The temperature is 10 °C; Table S3: The temperature is 16 °C; Table S4: The temperature is 20 °C; Table S5: The temperature is 25 °C; Table S6: The temperature is 30 °C.
