# Supplementary material for: Influence of Electrostatic Field on Optical Rotation of D-Glucose Solution: Experimental Research for Electric Field-Induced Biological Effect
Source: Molecules. 2024 Oct 16;29(20):4898. doi: 10.3390/molecules29204898 (PMC11510495; doi:10.3390/molecules29204898)
Supplement: Supplementary file 1 [file molecules-29-04898-s001.zip › molecules-3216898-supplementary.pdf]

Supplementary Materials:

Influence of Electrostatic Field on Optical Rotation of D-glucose Solution: Experimental Research for Electric Field-Induced Biological Effect

Quanlin Guo , Dezhi Gou *, Chenxi Zhao , Yun Ma , Chaojun Chen and Junxi Zhu

School of Electronic and Information Engineering, China West Normal University, Nanchong 637000, China; m18398999056@163.com (Q.G.); 15775649088@163.com (C.Z.); 17781805450@163.com (Y.M.); 15160321639@163.com (C.C.); 18284648157@163.com (J.Z.)

* Correspondence: gdz_scu@163.com

Table S1 The temperature is 5℃

Electric field intensity (V/m)	Parameter	1	2	3	4	5	6	7	8	9	10	11	12	13	14	15	Average	Standard deviation
31.25	α_E	0.264	0.263	0.262	0.261	0.261	0.262	0.264	0.260	0.261	0.261	0.262	0.262	0.263	0.262	0.261	0.262	0.001
	$[\alpha]_E$	52.837	52.574	52.564	52.338	52.238	52.443	52.512	52.191	52.227	52.373	52.383	52.574	52.584	52.465	52.330	52.442	0.172
	$C_{\alpha E}$	36.59%	36.31%	36.30%	36.05%	35.95%	36.17%	36.24%	35.90%	35.93%	36.09%	36.10%	36.31%	36.32%	36.19%	36.05%	36.17%	0.002
	$C_{\beta E}$	63.41%	63.69%	63.70%	63.95%	64.05%	63.83%	63.76%	64.10%	64.07%	63.91%	63.90%	63.69%	63.68%	63.81%	63.95%	63.83%	0.002
0	α_{no}	0.260	0.260	0.261	0.263	0.261	0.260	0.260	0.261	0.263	0.261	0.260	0.260	0.260	0.261	0.262	0.261	0.001
	$[\alpha]_{no}$	51.303	50.236	50.468	51.307	51.224	51.834	52.004	50.777	50.912	50.875	52.034	51.934	51.665	52.114	52.014	51.380	0.619
	$C_{\alpha no}$	34.94%	33.80%	34.05%	34.95%	34.86%	35.51%	35.70%	34.38%	34.53%	34.49%	35.73%	35.62%	35.33%	35.81%	35.71%	35.03%	0.007
	$C_{\beta no}$	65.06%	66.20%	65.95%	65.05%	65.14%	64.49%	64.30%	65.62%	65.47%	65.51%	64.27%	64.38%	64.67%	64.19%	64.29%	64.97%	0.007
	$(C_{\alpha E}-C_{\alpha no})/ C_{\alpha no}$	4.71%	7.41%	6.60%	3.16%	3.12%	1.84%	1.53%	4.41%	4.08%	4.66%	1.05%	1.93%	2.79%	1.05%	0.95%	3.25%	0.020
	$(C_{\beta E}-C_{\beta no})/ C_{\beta no}$	-2.53%	-3.79%	-3.41%	-1.70%	-1.67%	-1.01%	-0.85%	-2.31%	-2.15%	-2.45%	-0.58%	-1.07%	-1.52%	-0.59%	-0.53%	-1.75%	0.010
	$([\alpha]_E-[\alpha]_{no})/ [\alpha]_{no}$	2.99%	4.65%	4.15%	2.01%	1.98%	1.17%	0.98%	2.78%	2.58%	2.94%	0.67%	1.23%	1.78%	0.67%	0.61%	2.07%	0.013
62.5	α_E	0.275	0.278	0.273	0.272	0.274	0.266	0.265	0.265	0.266	0.264	0.267	0.266	0.266	0.265	0.267	0.269	0.004
	$[\alpha]_E$	54.483	55.494	54.510	54.170	55.055	53.066	53.315	53.169	53.246	52.733	53.535	53.432	53.393	53.410	53.666	53.778	0.788
	$C_{\alpha E}$	38.35%	39.44%	38.38%	38.02%	38.97%	36.83%	37.10%	36.94%	37.03%	36.48%	37.34%	37.23%	37.18%	37.20%	37.48%	37.60%	0.008
	$C_{\beta E}$	61.65%	60.56%	61.62%	61.98%	61.03%	63.17%	62.90%	63.06%	62.97%	63.52%	62.66%	62.77%	62.82%	62.80%	62.52%	62.40%	0.008
0	α_{no}	0.273	0.272	0.272	0.268	0.269	0.260	0.258	0.264	0.264	0.263	0.262	0.264	0.262	0.263	0.264	0.265	0.005
	$[\alpha]_{no}$	54.460	54.180	54.353	53.688	53.582	52.191	51.845	52.464	52.624	52.501	52.511	52.819	52.333	52.545	52.777	52.992	0.837
	$C_{\alpha no}$	38.33%	38.03%	38.21%	37.50%	37.39%	35.90%	35.53%	36.19%	36.36%	36.23%	36.24%	36.57%	36.05%	36.28%	36.52%	36.75%	0.009
	$C_{\beta no}$	61.67%	61.97%	61.79%	62.50%	62.61%	64.10%	64.47%	63.81%	63.64%	63.77%	63.76%	63.43%	63.95%	63.72%	63.48%	63.25%	0.009
	$(C_{\alpha E}-C_{\alpha no})/ C_{\alpha no}$	0.06%	3.70%	0.44%	1.38%	4.22%	2.61%	4.44%	2.09%	1.83%	0.69%	3.03%	1.80%	3.15%	2.56%	2.61%	2.29%	0.013
	$(C_{\beta E}-C_{\beta no})/ C_{\beta no}$	-0.04%	-2.27%	-0.27%	-0.83%	-2.52%	-1.46%	-2.44%	-1.18%	-1.05%	-0.39%	-1.72%	-1.04%	-1.78%	-1.45%	-1.50%	-1.33%	0.008
	$([\alpha]_E-[\alpha]_{no})/ [\alpha]_{no}$	0.04%	2.43%	0.29%	0.90%	2.75%	1.68%	2.84%	1.34%	1.18%	0.44%	1.95%	1.16%	2.03%	1.65%	1.68%	1.49%	0.008
125	α_E	0.272	0.271	0.268	0.268	0.266	0.271	0.269	0.274	0.273	0.271	0.270	0.268	0.269	0.269	0.270	0.270	0.002
	$[\alpha]_E$	53.682	54.164	53.692	53.385	53.083	54.583	53.648	54.443	54.593	53.854	53.933	53.812	54.017	54.116	54.231	53.949	0.426
	$C_{\alpha E}$	37.49%	38.01%	37.50%	37.18%	36.85%	38.46%	37.46%	38.31%	38.47%	37.68%	37.76%	37.63%	37.85%	37.96%	38.08%	37.78%	0.005
	$C_{\beta E}$	62.51%	61.99%	62.50%	62.82%	63.15%	61.54%	62.54%	61.69%	61.53%	62.32%	62.24%	62.37%	62.15%	62.04%	61.92%	62.22%	0.005
0	α_{no}	0.266	0.261	0.261	0.265	0.264	0.276	0.273	0.273	0.271	0.272	0.267	0.268	0.266	0.267	0.266	0.268	0.004
	$[\alpha]_{no}$	53.043	52.098	52.125	53.023	52.906	54.919	54.390	54.566	54.360	54.520	53.243	53.372	53.267	53.812	53.515	53.544	0.869
	$C_{\alpha no}$	36.81%	35.80%	35.83%	36.79%	36.66%	38.82%	38.25%	38.44%	38.22%	38.39%	37.02%	37.16%	37.05%	37.63%	37.32%	37.35%	0.009
	$C_{\beta no}$	63.19%	64.20%	64.17%	63.21%	63.34%	61.18%	61.75%	61.56%	61.78%	61.61%	62.98%	62.84%	62.95%	62.37%	62.68%	62.65%	0.009
	$(C_{\alpha E}-C_{\alpha no})/ C_{\alpha no}$	1.86%	6.19%	4.69%	1.05%	0.52%	-0.93%	-2.08%	-0.34%	0.65%	-1.86%	2.00%	1.27%	2.17%	0.87%	2.06%	1.16%	0.022
	$(C_{\beta E}-C_{\beta no})/ C_{\beta no}$	-1.08%	-3.45%	-2.62%	-0.61%	-0.30%	0.59%	1.29%	0.21%	-0.40%	1.16%	-1.17%	-0.75%	-1.28%	-0.52%	-1.22%	-0.69%	0.013
	$([\alpha]_E-[\alpha]_{no})/ [\alpha]_{no}$	1.20%	3.97%	3.01%	0.68%	0.33%	-0.61%	-1.36%	-0.23%	0.43%	-1.22%	1.30%	0.82%	1.41%	0.56%	1.34%	0.76%	0.014

Continuation of table S1 The temperature is 5℃

Electric field intensity (V/m)	Parameter	1	2	3	4	5	6	7	8	9	10	11	12	13	14	15	Average	Standard deviation
187.5	α_E	0.274	0.275	0.274	0.272	0.273	0.267	0.267	0.268	0.267	0.269	0.267	0.267	0.266	0.267	0.266	0.269	0.0032
	$[\alpha]_E$	55.017	55.141	55.236	54.833	55.001	53.681	53.558	53.808	53.522	53.748	53.131	53.412	52.936	53.440	53.386	53.990	0.7792
	$C_{\alpha E}$	38.92%	39.06%	39.16%	38.73%	38.91%	37.49%	37.36%	37.63%	37.32%	37.56%	36.90%	37.20%	36.69%	37.23%	37.18%	37.82%	0.0084
	$C_{\beta E}$	61.08%	60.94%	60.84%	61.27%	61.09%	62.51%	62.64%	62.37%	62.68%	62.44%	63.10%	62.80%	63.31%	62.77%	62.82%	62.18%	0.0084
0	α_{no}	0.272	0.273	0.272	0.273	0.274	0.266	0.262	0.261	0.264	0.263	0.262	0.262	0.264	0.262	0.263	0.266	0.0048
	$[\alpha]_{no}$	54.739	54.839	54.866	54.739	54.822	53.435	52.125	52.065	53.063	52.304	52.017	52.337	52.304	52.145	52.238	53.203	1.1870
	$C_{\alpha no}$	38.63%	38.73%	38.76%	38.63%	38.72%	37.23%	35.83%	35.76%	36.83%	36.02%	35.71%	36.05%	36.02%	35.85%	35.95%	36.98%	0.0127
	$C_{\beta no}$	61.37%	61.27%	61.24%	61.37%	61.28%	62.77%	64.17%	64.24%	63.17%	63.98%	64.29%	63.95%	63.98%	64.15%	64.05%	63.02%	0.0127
	$(C_{\alpha E}-C_{\alpha no})/C_{\alpha no}$	0.77%	0.84%	1.02%	0.26%	0.50%	0.71%	4.29%	5.22%	1.34%	4.30%	3.34%	3.20%	1.88%	3.87%	3.42%	2.28%	0.0162
	$(C_{\beta E}-C_{\beta no})/C_{\beta no}$	-0.49%	-0.53%	-0.65%	-0.16%	-0.31%	-0.42%	-2.39%	-2.91%	-0.78%	-2.42%	-1.86%	-1.80%	-1.06%	-2.16%	-1.92%	-1.34%	0.0089
	$([\alpha]_E-[\alpha]_{no})/[\alpha]_{no}$	0.51%	0.55%	0.67%	0.17%	0.33%	0.46%	2.75%	3.35%	0.87%	2.76%	2.14%	2.05%	1.21%	2.48%	2.20%	1.48%	0.0103
250	α_E	0.275	0.272	0.273	0.270	0.271	0.269	0.268	0.269	0.270	0.269	0.269	0.271	0.269	0.270	0.269	0.270	0.0018
	$[\alpha]_E$	55.055	54.437	54.290	54.353	54.526	53.936	54.001	54.137	54.212	53.867	53.854	54.312	54.016	53.939	53.837	54.185	0.3175
	$C_{\alpha E}$	38.97%	38.30%	38.15%	38.21%	38.40%	37.77%	37.84%	37.98%	38.06%	37.69%	37.68%	38.17%	37.85%	37.77%	37.66%	38.03%	0.0034
	$C_{\beta E}$	61.03%	61.70%	61.85%	61.79%	61.60%	62.23%	62.16%	62.02%	61.94%	62.31%	62.32%	61.83%	62.15%	62.23%	62.34%	61.97%	0.0034
0	α_{no}	0.274	0.275	0.273	0.275	0.272	0.267	0.268	0.267	0.267	0.267	0.265	0.266	0.266	0.266	0.267	0.269	0.0035
	$[\alpha]_{no}$	54.842	54.846	54.769	54.912	54.759	53.236	53.336	53.147	53.195	53.116	53.142	53.316	53.272	53.431	53.316	53.776	0.7473
	$C_{\alpha no}$	38.74%	38.74%	38.66%	38.81%	38.65%	37.02%	37.12%	36.92%	36.97%	36.89%	36.92%	37.10%	37.05%	37.23%	37.10%	37.59%	0.0080
	$C_{\beta no}$	61.26%	61.26%	61.34%	61.19%	61.35%	62.98%	62.88%	63.08%	63.03%	63.11%	63.08%	62.90%	62.95%	62.77%	62.90%	62.41%	0.0080
	$(C_{\alpha E}-C_{\alpha no})/C_{\alpha no}$	0.59%	-1.13%	-1.33%	-1.54%	-0.65%	2.03%	1.92%	2.87%	2.95%	2.18%	2.07%	2.88%	2.15%	1.46%	1.51%	1.17%	0.0154
	$(C_{\beta E}-C_{\beta no})/C_{\beta no}$	-0.37%	0.72%	0.84%	0.98%	0.41%	-1.19%	-1.13%	-1.68%	-1.73%	-1.28%	-1.21%	-1.70%	-1.27%	-0.87%	-0.89%	-0.70%	0.0093
	$([\alpha]_E-[\alpha]_{no})/[\alpha]_{no}$	0.39%	-0.75%	-0.87%	-1.02%	-0.43%	1.31%	1.25%	1.86%	1.91%	1.41%	1.34%	1.87%	1.40%	0.95%	0.98%	0.76%	0.0101

Comment: $C=\frac{[\alpha]-[\alpha]_\beta}{[\alpha]_\alpha}$, $C_{\alpha E}=\frac{[\alpha]_E-[\alpha]_\beta}{[\alpha]_\alpha}$, $C_{\alpha no}=\frac{[\alpha]_{no}-[\alpha]_\beta}{[\alpha]_\alpha}$, $C_{\beta E}=1-C_{\alpha E}$, $C_{\beta no}=1-C_{\alpha no}$, where $[\alpha]_\alpha$ is the intrinsic specific rotation of α-D glucose :+112°, $[\alpha]_\beta$ is the intrinsic specific rotation of β-D glucose :+18.7°, $C_{\alpha E}$ and $C_{\beta E}$ is proportion of α-D glucose and β-D glucose configuration in solution under electrostatic field, $C_{\alpha no}$ and $C_{\beta no}$ Proportion of α-D glucose and β-D glucose configuration in solution without electrostatic field, $[\alpha]_E$ is specific curl of D glucose under electrostatic field, $[\alpha]_{no}$ is specific curl of D glucose in the absence of electrostatic field. α_E is optical rotation of D glucose under electrostatic field, α_{no} is optical rotation of D glucose in the absence of electrostatic field.

Table S2 The temperature is 10℃

Electric field intensity (V/m)	Parameter	1	2	3	4	5	6	7	8	9	10	11	12	13	14	15	Average	Standard deviation
31.25	α_E	0.262	0.261	0.262	0.260	0.261	0.266	0.265	0.265	0.265	0.262	0.266	0.265	0.262	0.266	0.265	0.264	0.0021
	$[\alpha]_E$	52.504	52.055	52.567	52.869	52.421	53.291	53.146	52.907	53.003	52.507	53.311	52.979	53.011	53.217	53.144	52.862	0.3700
	$C_{\alpha E}$	36.23%	35.75%	36.30%	36.62%	36.14%	37.08%	36.92%	36.66%	36.77%	36.23%	37.10%	36.74%	36.77%	37.00%	36.92%	36.62%	0.0040
	$C_{\beta E}$	63.77%	64.25%	63.70%	63.38%	63.86%	62.92%	63.08%	63.34%	63.23%	63.77%	62.90%	63.26%	63.23%	63.00%	63.08%	63.38%	0.0040
0	α_{no}	0.266	0.268	0.267	0.267	0.267	0.262	0.260	0.263	0.263	0.264	0.263	0.262	0.262	0.263	0.263	0.264	0.0024
	$[\alpha]_{no}$	53.449	53.559	53.622	53.163	53.339	52.211	51.949	52.462	52.388	52.555	52.411	52.151	52.217	52.272	52.311	52.671	0.5788
	$C_{\alpha no}$	37.24%	37.36%	37.43%	36.94%	37.13%	35.92%	35.64%	36.19%	36.11%	36.29%	36.13%	35.85%	35.92%	35.98%	36.02%	36.41%	0.0062
	$C_{\beta no}$	62.76%	62.64%	62.57%	63.06%	62.87%	64.08%	64.36%	63.81%	63.89%	63.71%	63.87%	64.15%	64.08%	64.02%	63.98%	63.59%	0.0062
	$(C_{\alpha E}-C_{\alpha no})/ C_{\alpha no}$	-2.72%	-4.31%	-3.02%	-0.85%	-2.65%	3.22%	3.60%	1.32%	1.83%	-0.14%	2.67%	2.48%	2.37%	2.81%	2.48%	0.56%	0.0266
	$(C_{\beta E}-C_{\beta no})/ C_{\beta no}$	1.61%	2.57%	1.81%	0.50%	1.56%	-1.81%	-1.99%	-0.75%	-1.03%	0.08%	-1.51%	-1.38%	-1.33%	-1.58%	-1.40%	-0.32%	0.0154
	$([\alpha]_E-[\alpha]_{no})/ [\alpha]_{no}$	-1.77%	-2.81%	-1.97%	-0.55%	-1.72%	2.07%	2.30%	0.85%	1.17%	-0.09%	1.72%	1.59%	1.52%	1.81%	1.59%	0.36%	0.0172
62.5	α_E	0.261	0.262	0.262	0.260	0.263	0.275	0.275	0.273	0.274	0.274	0.272	0.273	0.272	0.271	0.273	0.269	0.0058
	$[\alpha]_E$	52.577	52.182	52.311	51.982	52.451	54.816	54.879	54.463	54.410	54.862	54.211	54.194	53.955	53.876	54.011	53.679	1.0613
	$C_{\alpha E}$	36.31%	35.89%	36.02%	35.67%	36.17%	38.71%	38.78%	38.33%	38.27%	38.76%	38.06%	38.04%	37.79%	37.70%	37.85%	37.49%	0.0114
	$C_{\beta E}$	63.69%	64.11%	63.98%	64.33%	63.83%	61.29%	61.22%	61.67%	61.73%	61.24%	61.94%	61.96%	62.21%	62.30%	62.15%	62.51%	0.0114
0	α_{no}	0.268	0.264	0.264	0.264	0.265	0.270	0.269	0.269	0.268	0.268	0.266	0.267	0.266	0.266	0.267	0.267	0.0019
	$[\alpha]_{no}$	53.302	52.737	52.843	52.963	52.913	54.180	53.855	53.681	53.768	53.666	53.151	53.259	53.117	53.363	53.333	53.342	0.4145
	$C_{\alpha no}$	37.09%	36.48%	36.59%	36.72%	36.67%	38.03%	37.68%	37.49%	37.59%	37.48%	36.92%	37.04%	36.89%	37.15%	37.12%	37.13%	0.0044
	$C_{\beta no}$	62.91%	63.52%	63.41%	63.28%	63.33%	61.97%	62.32%	62.51%	62.41%	62.52%	63.08%	62.96%	63.11%	62.85%	62.88%	62.87%	0.0044
	$(C_{\alpha E}-C_{\alpha no})/ C_{\alpha no}$	-2.10%	-1.63%	-1.56%	-2.86%	-1.35%	1.79%	2.91%	2.24%	1.83%	3.42%	3.08%	2.71%	2.43%	1.48%	1.96%	0.97%	0.0218
	$(C_{\beta E}-C_{\beta no})/ C_{\beta no}$	1.24%	0.94%	0.90%	1.66%	0.78%	-1.10%	-1.76%	-1.34%	-1.10%	-2.05%	-1.80%	-1.59%	-1.42%	-0.87%	-1.16%	-0.57%	0.0128
	$([\alpha]_E-[\alpha]_{no})/ [\alpha]_{no}$	-1.36%	-1.05%	-1.01%	-1.85%	-0.87%	1.17%	1.90%	1.46%	1.19%	2.23%	1.99%	1.76%	1.58%	0.96%	1.27%	0.63%	0.0141
125	α_E	0.262	0.265	0.263	0.265	0.263	0.268	0.266	0.265	0.265	0.266	0.262	0.260	0.261	0.261	0.262	0.264	0.0023
	$[\alpha]_E$	52.345	53.144	52.540	52.944	52.744	53.608	53.242	53.100	53.063	53.226	52.444	52.016	52.357	52.266	52.123	52.744	0.4811
	$C_{\alpha E}$	36.06%	36.92%	36.27%	36.70%	36.49%	37.41%	37.02%	36.87%	36.83%	37.01%	36.17%	35.71%	36.07%	35.98%	35.82%	36.49%	0.0052
	$C_{\beta E}$	63.94%	63.08%	63.73%	63.30%	63.51%	62.59%	62.98%	63.13%	63.17%	62.99%	63.83%	64.29%	63.93%	64.02%	64.18%	63.51%	0.0052
0	α_{no}	0.262	0.263	0.262	0.263	0.262	0.265	0.264	0.264	0.264	0.263	0.261	0.261	0.260	0.261	0.261	0.262	0.0015
	$[\alpha]_{no}$	52.406	52.546	52.376	52.576	52.476	52.993	52.750	52.873	52.863	52.727	52.147	52.116	52.006	52.131	52.159	52.476	0.3186
	$C_{\alpha no}$	36.13%	36.28%	36.09%	36.31%	36.20%	36.76%	36.50%	36.63%	36.62%	36.47%	35.85%	35.82%	35.70%	35.83%	35.86%	36.20%	0.0034
	$C_{\beta no}$	63.87%	63.72%	63.91%	63.69%	63.80%	63.24%	63.50%	63.37%	63.38%	63.53%	64.15%	64.18%	64.30%	64.17%	64.14%	63.80%	0.0034
	$(C_{\alpha E}-C_{\alpha no})/ C_{\alpha no}$	-0.18%	1.77%	0.49%	1.09%	0.79%	1.79%	1.44%	0.66%	0.59%	1.47%	0.89%	-0.30%	1.05%	0.40%	-0.11%	0.79%	0.0067
	$(C_{\beta E}-C_{\beta no})/ C_{\beta no}$	0.10%	-1.01%	-0.28%	-0.62%	-0.45%	-1.04%	-0.83%	-0.38%	-0.34%	-0.84%	-0.50%	0.17%	-0.59%	-0.23%	0.06%	-0.45%	0.0038
	$([\alpha]_E-[\alpha]_{no})/ [\alpha]_{no}$	-0.12%	1.14%	0.31%	0.70%	0.51%	1.16%	0.93%	0.43%	0.38%	0.95%	0.57%	-0.19%	0.67%	0.26%	-0.07%	0.51%	0.0043

Continuation of table S2 The temperature is 10℃

Electric field intensity (V/m)	Parameter	1	2	3	4	5	6	7	8	9	10	11	12	13	14	15	Average	Standard deviation
187.5	α_E	0.270	0.270	0.271	0.270	0.271	0.257	0.258	0.259	0.256	0.256	0.263	0.262	0.263	0.263	0.262	0.263	0.0057
	$[\alpha]_E$	53.998	54.001	53.961	54.340	54.133	51.523	51.486	51.696	51.100	51.194	52.534	52.211	52.444	52.567	52.151	52.623	1.1640
	$C_{\alpha E}$	37.83%	37.84%	37.79%	38.20%	37.98%	35.18%	35.14%	35.37%	34.73%	34.83%	36.26%	35.92%	36.17%	36.30%	35.85%	36.36%	0.0125
	$C_{\beta E}$	62.17%	62.16%	62.21%	61.80%	62.02%	64.82%	64.86%	64.63%	65.27%	65.17%	63.74%	64.08%	63.83%	63.70%	64.15%	63.64%	0.0125
0	α_{no}	0.274	0.270	0.272	0.270	0.270	0.255	0.254	0.256	0.253	0.253	0.262	0.260	0.262	0.261	0.262	0.262	0.0073
	$[\alpha]_{no}$	54.333	54.084	54.360	54.120	54.021	50.515	50.495	50.475	50.508	50.349	52.257	51.987	52.177	52.014	52.273	52.265	1.5767
	$C_{\alpha no}$	38.19%	37.92%	38.22%	37.96%	37.86%	34.10%	34.08%	34.06%	34.09%	33.92%	35.97%	35.68%	35.88%	35.71%	35.98%	35.97%	0.0169
	$C_{\beta no}$	61.81%	62.08%	61.78%	62.04%	62.14%	65.90%	65.92%	65.94%	65.91%	66.08%	64.03%	64.32%	64.12%	64.29%	64.02%	64.03%	0.0169
	$(C_{\alpha E}-C_{\alpha no})/ C_{\alpha no}$	-0.94%	-0.23%	-1.12%	0.62%	0.32%	3.17%	3.12%	3.84%	1.86%	2.67%	0.83%	0.67%	0.80%	1.66%	-0.36%	1.07%	0.0154
	$(C_{\beta E}-C_{\beta no})/ C_{\beta no}$	0.58%	0.14%	0.69%	-0.38%	-0.19%	-1.64%	-1.61%	-1.98%	-0.96%	-1.37%	-0.46%	-0.37%	-0.45%	-0.92%	0.20%	-0.60%	0.0082
	$([\alpha]_E-[\alpha]_{no})/ [\alpha]_{no}$	-0.62%	-0.15%	-0.73%	0.41%	0.21%	2.00%	1.96%	2.42%	1.17%	1.68%	0.53%	0.43%	0.51%	1.06%	-0.23%	0.69%	0.0098
250	α_E	0.264	0.264	0.263	0.264	0.265	0.264	0.263	0.264	0.261	0.263	0.264	0.263	0.263	0.261	0.262	0.263	0.0011
	$[\alpha]_E$	52.500	52.902	52.652	52.750	52.702	53.365	52.773	52.451	52.816	52.644	52.898	52.635	52.667	52.234	52.535	52.702	0.2536
	$C_{\alpha E}$	36.23%	36.66%	36.39%	36.50%	36.44%	37.15%	36.52%	36.17%	36.57%	36.38%	36.65%	36.37%	36.41%	35.94%	36.26%	36.44%	0.0027
	$C_{\beta E}$	63.77%	63.34%	63.61%	63.50%	63.56%	62.85%	63.48%	63.83%	63.43%	63.62%	63.35%	63.63%	63.59%	64.06%	63.74%	63.56%	0.0027
0	α_{no}	0.266	0.267	0.261	0.262	0.263	0.260	0.262	0.262	0.261	0.262	0.260	0.262	0.261	0.261	0.261	0.262	0.0020
	$[\alpha]_{no}$	52.175	52.579	52.217	52.530	52.377	52.943	52.565	52.185	52.301	52.557	52.134	52.363	52.344	52.267	52.118	52.377	0.2237
	$C_{\alpha no}$	35.88%	36.31%	35.92%	36.26%	36.10%	36.70%	36.30%	35.89%	36.01%	36.29%	35.83%	36.08%	36.06%	35.98%	35.82%	36.10%	0.0024
	$C_{\beta no}$	64.12%	63.69%	64.08%	63.74%	63.90%	63.30%	63.70%	64.11%	63.99%	63.71%	64.17%	63.92%	63.94%	64.02%	64.18%	63.90%	0.0024
	$(C_{\alpha E}-C_{\alpha no})/ C_{\alpha no}$	0.97%	0.95%	1.30%	0.65%	0.97%	1.23%	0.61%	0.79%	1.53%	0.26%	2.29%	0.81%	0.96%	-0.10%	1.25%	0.96%	0.0055
	$(C_{\beta E}-C_{\beta no})/ C_{\beta no}$	-0.54%	-0.54%	-0.73%	-0.37%	-0.55%	-0.71%	-0.35%	-0.44%	-0.86%	-0.15%	-1.28%	-0.46%	-0.54%	0.06%	-0.70%	-0.54%	0.0031
	$([\alpha]_E-[\alpha]_{no})/ [\alpha]_{no}$	0.62%	0.61%	0.83%	0.42%	0.62%	0.80%	0.40%	0.51%	0.98%	0.17%	1.47%	0.52%	0.62%	-0.06%	0.80%	0.62%	0.0035

Table S3 The temperature is 16℃

Electric field intensity (V/m)	Parameter	1	2	3	4	5	6	7	8	9	10	11	12	13	14	15	Average	Standard deviation
31.25	α_E	0.276	0.275	0.276	0.275	0.276	0.278	0.278	0.276	0.279	0.280	0.280	0.279	0.270	0.273	0.277	0.277	0.0027
	$[\alpha]_E$	55.275	55.079	55.217	55.149	55.333	55.683	55.879	55.741	55.479	55.986	56.106	56.173	55.824	54.014	54.776	55.448	0.5699
	$C_{\alpha E}$	39.20%	38.99%	39.14%	39.07%	39.26%	39.64%	39.85%	39.70%	39.42%	39.96%	40.09%	40.16%	39.79%	37.85%	38.67%	39.39%	0.0061
	$C_{\beta E}$	60.80%	61.01%	60.86%	60.93%	60.74%	60.36%	60.15%	60.30%	60.58%	60.04%	59.91%	59.84%	60.21%	62.15%	61.33%	60.61%	0.0061
0	α_{no}	0.275	0.274	0.275	0.275	0.276	0.276	0.276	0.275	0.278	0.280	0.276	0.279	0.275	0.275	0.275	0.276	0.0017
	$[\alpha]_{no}$	55.073	54.993	55.102	55.101	55.174	55.310	55.300	55.284	55.193	55.76	56.186	55.308	55.863	54.054	53.991	55.179	0.5741
	$C_{\alpha no}$	38.98%	38.90%	39.02%	39.02%	39.09%	39.24%	39.23%	39.21%	39.11%	39.72%	40.18%	39.24%	39.83%	37.89%	37.83%	39.10%	0.0062
	$C_{\beta no}$	61.02%	61.10%	60.98%	60.98%	60.91%	60.76%	60.77%	60.79%	60.89%	60.28%	59.82%	60.76%	60.17%	62.11%	62.17%	60.90%	0.0062
	$(C_{\alpha E}-C_{\alpha no})/ C_{\alpha no}$	0.56%	0.24%	0.32%	0.13%	0.44%	1.02%	1.58%	1.25%	0.78%	0.61%	-0.21%	2.36%	-0.10%	-0.11%	2.22%	0.74%	0.0081
	$(C_{\beta E}-C_{\beta no})/ C_{\beta no}$	-0.35%	-0.15%	-0.20%	-0.08%	-0.28%	-0.66%	-1.02%	-0.81%	-0.50%	-0.40%	0.14%	-1.53%	0.07%	0.07%	-1.35%	-0.47%	0.0051
	$([\alpha]_E-[\alpha]_{no})/ [\alpha]_{no}$	0.37%	0.16%	0.21%	0.09%	0.29%	0.67%	1.05%	0.83%	0.52%	0.41%	-0.14%	1.56%	-0.07%	-0.07%	1.45%	0.49%	0.0053
62.5	α_E	0.273	0.276	0.273	0.272	0.277	0.269	0.270	0.270	0.270	0.270	0.275	0.277	0.275	0.274	0.276	0.273	0.0028
	$[\alpha]_E$	54.719	55.288	54.742	54.5	55.211	53.795	53.731	53.974	53.964	53.917	55.238	55.498	54.616	54.792	54.026	54.534	0.6019
	$C_{\alpha E}$	38.61%	39.22%	38.63%	38.37%	39.13%	37.62%	37.55%	37.81%	37.80%	37.75%	39.16%	39.44%	38.50%	38.68%	37.86%	38.41%	0.0065
	$C_{\beta E}$	61.39%	60.78%	61.37%	61.63%	60.87%	62.38%	62.45%	62.19%	62.20%	62.25%	60.84%	60.56%	61.50%	61.32%	62.14%	61.59%	0.0065
0	α_{no}	0.270	0.271	0.269	0.271	0.271	0.270	0.270	0.269	0.268	0.268	0.278	0.279	0.278	0.277	0.276	0.272	0.0040
	$[\alpha]_{no}$	54.12	54.087	53.575	53.834	53.984	54.094	53.894	53.568	53.688	53.672	55.694	55.634	55.328	55.395	53.671	54.283	0.7929
	$C_{\alpha no}$	37.96%	37.93%	37.38%	37.66%	37.82%	37.94%	37.72%	37.37%	37.50%	37.48%	39.65%	39.59%	39.26%	39.33%	37.48%	38.14%	0.0085
	$C_{\beta no}$	62.04%	62.07%	62.62%	62.34%	62.18%	62.06%	62.28%	62.63%	62.50%	62.52%	60.35%	60.41%	60.74%	60.67%	62.52%	61.86%	0.0085
	$(C_{\alpha E}-C_{\alpha no})/ C_{\alpha no}$	1.69%	3.39%	3.35%	1.90%	3.48%	-0.84%	-0.46%	1.16%	0.79%	0.70%	-1.23%	-0.37%	-1.94%	-1.64%	1.01%	0.71%	0.0181
	$(C_{\beta E}-C_{\beta no})/ C_{\beta no}$	-1.03%	-2.07%	-2.00%	-1.14%	-2.11%	0.52%	0.28%	-0.69%	-0.47%	-0.42%	0.81%	0.24%	1.26%	1.07%	-0.61%	-0.44%	0.0112
	$([\alpha]_E-[\alpha]_{no})/ [\alpha]_{no}$	1.11%	2.22%	2.18%	1.24%	2.27%	-0.55%	-0.30%	0.76%	0.51%	0.46%	-0.82%	-0.24%	-1.29%	-1.09%	0.66%	0.46%	0.0119
125	α_E	0.274	0.274	0.273	0.274	0.275	0.275	0.274	0.275	0.275	0.275	0.275	0.276	0.275	0.275	0.275	0.275	0.0007
	$[\alpha]_E$	54.895	54.957	54.799	54.998	55.014	55.011	54.858	55.16	55.024	55.15	55.091	55.249	55.05	55.034	55.037	55.022	0.1167
	$C_{\alpha E}$	38.79%	38.86%	38.69%	38.90%	38.92%	38.92%	38.75%	39.08%	38.93%	39.07%	39.00%	39.17%	38.96%	38.94%	38.95%	38.93%	0.0013
	$C_{\beta E}$	61.21%	61.14%	61.31%	61.10%	61.08%	61.08%	61.25%	60.92%	61.07%	60.93%	61.00%	60.83%	61.04%	61.06%	61.05%	61.07%	0.0013
0	α_{no}	0.273	0.273	0.273	0.272	0.271	0.272	0.273	0.275	0.273	0.274	0.273	0.274	0.275	0.276	0.273	0.273	0.0013
	$[\alpha]_{no}$	54.704	54.7	54.763	54.535	54.234	54.315	54.556	55.077	54.792	54.896	54.733	54.961	55.181	55.262	54.611	54.755	0.2910
	$C_{\alpha no}$	38.59%	38.59%	38.65%	38.41%	38.09%	38.17%	38.43%	38.99%	38.68%	38.80%	38.62%	38.86%	39.10%	39.19%	38.49%	38.64%	0.0031
	$C_{\beta no}$	61.41%	61.41%	61.35%	61.59%	61.91%	61.83%	61.57%	61.01%	61.32%	61.20%	61.38%	61.14%	60.90%	60.81%	61.51%	61.36%	0.0031
	$(C_{\alpha E}-C_{\alpha no})/ C_{\alpha no}$	0.53%	0.71%	0.10%	1.29%	2.20%	1.95%	0.84%	0.23%	0.64%	0.70%	0.99%	0.79%	-0.36%	-0.62%	1.19%	0.74%	0.0075
	$(C_{\beta E}-C_{\beta no})/ C_{\beta no}$	-0.33%	-0.45%	-0.06%	-0.81%	-1.35%	-1.21%	-0.53%	-0.15%	-0.41%	-0.44%	-0.63%	-0.50%	0.23%	0.40%	-0.74%	-0.47%	0.0047
	$([\alpha]_E-[\alpha]_{no})/ [\alpha]_{no}$	0.35%	0.47%	0.07%	0.85%	1.44%	1.28%	0.55%	0.15%	0.42%	0.46%	0.65%	0.52%	-0.24%	-0.41%	0.78%	0.49%	0.0050

Continuation of table S3 The temperature is 16℃

Electric field intensity (V/m)	Parameter	1	2	3	4	5	6	7	8	9	10	11	12	13	14	15	Average	Standard deviation
187.5	α_E	0.261	0.265	0.266	0.269	0.266	0.271	0.270	0.268	0.271	0.270	0.273	0.268	0.271	0.269	0.266	0.268	0.0031
	$[\alpha]_E$	52.391	53.149	53.399	53.95	53.256	54.239	54.017	53.625	54.157	54.187	54.619	53.652	54.387	53.931	53.562	53.768	0.5689
	$C_{\alpha E}$	36.11%	36.92%	37.19%	37.78%	37.04%	38.09%	37.85%	37.43%	38.00%	38.04%	38.50%	37.46%	38.25%	37.76%	37.37%	37.59%	0.0061
	$C_{\beta E}$	63.89%	63.08%	62.81%	62.22%	62.96%	61.91%	62.15%	62.57%	62.00%	61.96%	61.50%	62.54%	61.75%	62.24%	62.63%	62.41%	0.0061
0	α_{no}	0.267	0.267	0.266	0.267	0.267	0.269	0.270	0.271	0.270	0.268	0.268	0.263	0.269	0.261	0.267	0.267	0.0026
	$[\alpha]_{no}$	53.502	53.422	53.289	53.465	53.492	53.934	54.021	54.237	54.057	53.864	53.781	52.614	53.801	52.255	53.419	53.544	0.5332
	$C_{\alpha no}$	37.30%	37.22%	37.07%	37.26%	37.29%	37.76%	37.86%	38.09%	37.90%	37.69%	37.60%	36.35%	37.62%	35.96%	37.21%	37.35%	0.0057
	$C_{\beta no}$	62.70%	62.78%	62.93%	62.74%	62.71%	62.24%	62.14%	61.91%	62.10%	62.31%	62.40%	63.65%	62.38%	64.04%	62.79%	62.65%	0.0057
	$(C_{\alpha E}-C_{\alpha no})/ C_{\alpha no}$	-3.19%	-0.79%	0.32%	1.40%	-0.68%	0.87%	-0.01%	-1.72%	0.28%	0.92%	2.39%	3.06%	1.67%	4.99%	0.41%	0.64%	0.0197
	$(C_{\beta E}-C_{\beta no})/ C_{\beta no}$	1.90%	0.47%	-0.19%	-0.83%	0.40%	-0.53%	0.01%	1.06%	-0.17%	-0.56%	-1.44%	-1.75%	-1.01%	-2.81%	-0.24%	-0.38%	0.0115
	$([\alpha]_E-[\alpha]_{no})/ [\alpha]_{no}$	-2.08%	-0.51%	0.21%	0.91%	-0.44%	0.57%	-0.01%	-1.13%	0.18%	0.60%	1.56%	1.97%	1.09%	3.21%	0.27%	0.42%	0.0128
250	α_E	0.266	0.266	0.266	0.267	0.265	0.274	0.275	0.275	0.274	0.275	0.272	0.272	0.271	0.271	0.268	0.270	0.0038
	$[\alpha]_E$	53.252	53.259	53.275	53.352	53.139	54.936	55.112	55.131	54.816	55.404	54.44	54.49	54.273	54.227	53.661	54.184	0.8013
	$C_{\alpha E}$	37.03%	37.04%	37.06%	37.14%	36.91%	38.84%	39.03%	39.05%	38.71%	39.34%	38.31%	38.36%	38.13%	38.08%	37.47%	38.03%	0.0086
	$C_{\beta E}$	62.97%	62.96%	62.94%	62.86%	63.09%	61.16%	60.97%	60.95%	61.29%	60.66%	61.69%	61.64%	61.87%	61.92%	62.53%	61.97%	0.0086
0	α_{no}	0.267	0.265	0.267	0.266	0.265	0.265	0.265	0.263	0.265	0.266	0.266	0.265	0.265	0.261	0.263	0.265	0.0016
	$[\alpha]_{no}$	53.795	53.146	53.562	53.296	53.123	53.159	53.12	52.757	52.927	52.564	53.233	52.97	53.166	53.249	52.796	53.124	0.3072
	$C_{\alpha no}$	37.62%	36.92%	37.37%	37.08%	36.89%	36.93%	36.89%	36.50%	36.68%	36.30%	37.01%	36.73%	36.94%	37.03%	36.54%	36.90%	0.0033
	$C_{\beta no}$	62.38%	63.08%	62.63%	62.92%	63.11%	63.07%	63.11%	63.50%	63.32%	63.70%	62.99%	63.27%	63.06%	62.97%	63.46%	63.10%	0.0033
	$(C_{\alpha E}-C_{\alpha no})/ C_{\alpha no}$	-1.55%	0.33%	-0.82%	0.16%	0.05%	5.16%	5.79%	6.97%	5.52%	8.39%	3.50%	4.44%	3.21%	2.83%	2.54%	3.08%	0.0299
	$(C_{\beta E}-C_{\beta no})/ C_{\beta no}$	0.93%	-0.19%	0.49%	-0.10%	-0.03%	-3.02%	-3.38%	-4.01%	-3.20%	-4.78%	-2.05%	-2.57%	-1.88%	-1.66%	-1.46%	-1.80%	0.0173
	$([\alpha]_E-[\alpha]_{no})/ [\alpha]_{no}$	-1.01%	0.21%	-0.54%	0.11%	0.03%	3.34%	3.75%	4.50%	3.57%	5.40%	2.27%	2.87%	2.08%	1.84%	1.64%	2.00%	0.0193

Table S4 The temperature is 20℃

Electric field intensity (V/m)	Parameter	1	2	3	4	5	6	7	8	9	10	11	12	13	14	15	Average	Standard deviation
31.25	α_E	0.263	0.262	0.263	0.262	0.263	0.262	0.261	0.260	0.261	0.260	0.279	0.278	0.277	0.276	0.278	0.267	0.0078
	$[\alpha]_E$	52.61	52.364	52.388	52.284	52.281	52.611	52.042	52.048	52.304	51.902	55.833	55.78	55.494	55.241	53.869	53.270	1.5185
	$C_{\alpha E}$	36.35%	36.08%	36.11%	36.00%	35.99%	36.35%	35.74%	35.74%	36.02%	35.59%	39.80%	39.74%	39.44%	39.17%	37.69%	37.05%	0.0163
	$C_{\beta E}$	63.65%	63.92%	63.89%	64.00%	64.01%	63.65%	64.26%	64.26%	63.98%	64.41%	60.20%	60.26%	60.56%	60.83%	62.31%	62.95%	0.0163
0	α_{no}	0.257	0.256	0.257	0.256	0.257	0.257	0.257	0.258	0.257	0.257	0.275	0.274	0.274	0.275	0.274	0.263	0.0086
	$[\alpha]_{no}$	51.433	51.247	51.187	50.967	51.124	51.633	51.406	51.466	51.473	51.526	54.939	54.879	54.939	54.892	53.347	52.431	1.6393
	$C_{\alpha no}$	35.08%	34.88%	34.82%	34.58%	34.75%	35.30%	35.05%	35.12%	35.13%	35.18%	38.84%	38.78%	38.84%	38.79%	37.14%	36.15%	0.0176
	$C_{\beta no}$	64.92%	65.12%	65.18%	65.42%	65.25%	64.70%	64.95%	64.88%	64.87%	64.82%	61.16%	61.22%	61.16%	61.21%	62.86%	63.85%	0.0176
	$(C_{\alpha E}-C_{\alpha no})/ C_{\alpha no}$	3.60%	3.43%	3.70%	4.08%	3.57%	2.97%	1.94%	1.78%	2.54%	1.15%	2.47%	2.49%	1.53%	0.96%	1.51%	2.49%	0.0101
	$(C_{\beta E}-C_{\beta no})/ C_{\beta no}$	-1.94%	-1.84%	-1.97%	-2.16%	-1.90%	-1.62%	-1.05%	-0.96%	-1.37%	-0.62%	-1.57%	-1.58%	-0.97%	-0.61%	-0.89%	-1.41%	0.0052
	$([\alpha]_E-[\alpha]_{no})/ [\alpha]_{no}$	2.29%	2.18%	2.35%	2.58%	2.26%	1.89%	1.24%	1.13%	1.61%	0.73%	1.63%	1.64%	1.01%	0.64%	0.98%	1.60%	0.0063
62.5	α_E	0.266	0.266	0.266	0.266	0.267	0.276	0.275	0.276	0.277	0.276	0.266	0.266	0.266	0.267	0.266	0.269	0.0048
	$[\alpha]_E$	53.145	53.072	53.185	52.998	53.145	54.949	54.959	55.078	55.255	55.178	53.316	53.402	53.159	53.322	52.900	53.804	0.9473
	$C_{\alpha E}$	36.92%	36.84%	36.96%	36.76%	36.92%	38.85%	38.86%	38.99%	39.18%	39.10%	37.10%	37.19%	36.93%	37.11%	36.66%	37.63%	0.0102
	$C_{\beta E}$	63.08%	63.16%	63.04%	63.24%	63.08%	61.15%	61.14%	61.01%	60.82%	60.90%	62.90%	62.81%	63.07%	62.89%	63.34%	62.37%	0.0102
0	α_{no}	0.266	0.262	0.262	0.263	0.264	0.269	0.269	0.268	0.269	0.268	0.262	0.264	0.263	0.264	0.265	0.265	0.0027
	$[\alpha]_{no}$	53.053	52.451	52.404	52.743	52.750	53.841	53.801	53.775	53.768	53.452	52.460	52.713	52.688	52.748	52.868	53.034	0.5389
	$C_{\alpha no}$	36.82%	36.17%	36.12%	36.49%	36.50%	37.66%	37.62%	37.59%	37.59%	37.25%	36.18%	36.46%	36.43%	36.49%	36.62%	36.80%	0.0058
	$C_{\beta no}$	63.18%	63.83%	63.88%	63.51%	63.50%	62.34%	62.38%	62.41%	62.41%	62.75%	63.82%	63.54%	63.57%	63.51%	63.38%	63.20%	0.0058
	$(C_{\alpha E}-C_{\alpha no})/ C_{\alpha no}$	0.27%	1.84%	2.32%	0.75%	1.16%	3.15%	3.30%	3.71%	4.24%	4.97%	2.54%	2.03%	1.39%	1.69%	0.09%	2.24%	0.0143
	$(C_{\beta E}-C_{\beta no})/ C_{\beta no}$	-0.16%	-1.04%	-1.31%	-0.43%	-0.67%	-1.91%	-1.99%	-2.24%	-2.55%	-2.95%	-1.44%	-1.16%	-0.79%	-0.97%	-0.05%	-1.31%	0.0087
	$([\alpha]_E-[\alpha]_{no})/ [\alpha]_{no}$	0.17%	1.18%	1.49%	0.48%	0.75%	2.06%	2.15%	2.42%	2.77%	3.23%	1.63%	1.31%	0.89%	1.09%	0.06%	1.45%	0.0094
125	α_E	0.274	0.274	0.273	0.274	0.275	0.275	0.274	0.275	0.275	0.275	0.275	0.276	0.275	0.275	0.275	0.275	0.0007
	$[\alpha]_E$	54.612	55.356	54.704	55.264	54.984	54.749	55.321	55.052	54.749	55.058	55.032	54.957	55.148	55.014	54.768	54.985	0.2307
	$C_{\alpha E}$	38.49%	39.29%	38.59%	39.19%	38.89%	38.64%	39.25%	38.96%	38.64%	38.97%	38.94%	38.86%	39.07%	38.92%	38.66%	38.89%	0.0025
	$C_{\beta E}$	61.51%	60.71%	61.41%	60.81%	61.11%	61.36%	60.75%	61.04%	61.36%	61.03%	61.06%	61.14%	60.93%	61.08%	61.34%	61.11%	0.0025
0	α_{no}	0.273	0.273	0.273	0.272	0.271	0.272	0.273	0.275	0.273	0.274	0.273	0.274	0.275	0.276	0.273	0.273	0.0013
	$[\alpha]_{no}$	54.502	55.100	54.611	54.993	54.802	54.158	54.939	54.939	55.038	54.879	54.742	54.858	54.892	54.771	54.812	54.802	0.2363
	$C_{\alpha no}$	38.37%	39.01%	38.49%	38.90%	38.69%	38.00%	38.84%	38.84%	38.95%	38.78%	38.63%	38.75%	38.79%	38.66%	38.71%	38.69%	0.0025
	$C_{\beta no}$	61.63%	60.99%	61.51%	61.10%	61.31%	62.00%	61.16%	61.16%	61.05%	61.22%	61.37%	61.25%	61.21%	61.34%	61.29%	61.31%	0.0025
	$(C_{\alpha E}-C_{\alpha no})/ C_{\alpha no}$	0.31%	0.70%	0.26%	0.75%	0.50%	1.67%	1.05%	0.31%	-0.80%	0.49%	0.80%	0.27%	0.71%	0.67%	-0.12%	0.50%	0.0055
	$(C_{\beta E}-C_{\beta no})/ C_{\beta no}$	-0.19%	-0.45%	-0.16%	-0.48%	-0.32%	-1.02%	-0.67%	-0.20%	0.51%	-0.31%	-0.51%	-0.17%	-0.45%	-0.42%	0.08%	-0.32%	0.0034
	$([\alpha]_E-[\alpha]_{no})/ [\alpha]_{no}$	0.20%	0.46%	0.17%	0.49%	0.33%	1.09%	0.70%	0.21%	-0.53%	0.33%	0.53%	0.18%	0.47%	0.44%	-0.08%	0.33%	0.0036

Continuation of table S4 The temperature is 20℃

Electric field intensity (V/m)	Parameter	1	2	3	4	5	6	7	8	9	10	11	12	13	14	15	Average	Standard deviation
187.5	α_E	0.271	0.271	0.270	0.269	0.271	0.265	0.267	0.267	0.267	0.265	0.267	0.266	0.267	0.268	0.266	0.268	0.0021
	$[\alpha]_E$	53.983	54.237	54.011	54.209	54.11	53.186	53.143	53.399	53.226	52.943	53.442	53.352	53.262	53.243	53.502	53.550	0.4346
	$C_{\alpha E}$	37.82%	38.09%	37.85%	38.06%	37.95%	36.96%	36.92%	37.19%	37.01%	36.70%	37.24%	37.14%	37.04%	37.02%	37.30%	37.35%	0.0047
	$C_{\beta E}$	62.18%	61.91%	62.15%	61.94%	62.05%	63.04%	63.08%	62.81%	62.99%	63.30%	62.76%	62.86%	62.96%	62.98%	62.70%	62.65%	0.0047
0	α_{no}	0.272	0.271	0.270	0.270	0.270	0.262	0.262	0.261	0.262	0.260	0.260	0.259	0.263	0.262	0.262	0.264	0.0047
	$[\alpha]_{no}$	54.26	54.3	53.961	53.958	54.071	52.321	52.397	52.324	52.484	52.324	52.291	51.992	52.254	52.404	52.344	52.912	0.8871
	$C_{\alpha no}$	38.11%	38.16%	37.79%	37.79%	37.91%	36.04%	36.12%	36.04%	36.21%	36.04%	36.00%	35.68%	35.96%	36.12%	36.06%	36.67%	0.0095
	$C_{\beta no}$	61.89%	61.84%	62.21%	62.21%	62.09%	63.96%	63.88%	63.96%	63.79%	63.96%	64.00%	64.32%	64.04%	63.88%	63.94%	63.33%	0.0095
	$(C_{\alpha E}-C_{\alpha no})/ C_{\alpha no}$	-0.78%	-0.18%	0.14%	0.71%	0.11%	2.57%	2.21%	3.20%	2.20%	1.84%	3.43%	4.09%	3.00%	2.49%	3.44%	1.86%	0.0153
	$(C_{\beta E}-C_{\beta no})/ C_{\beta no}$	0.48%	0.11%	-0.09%	-0.43%	-0.07%	-1.45%	-1.25%	-1.80%	-1.25%	-1.04%	-1.93%	-2.27%	-1.69%	-1.41%	-1.94%	-1.08%	0.0086
	$([\alpha]_E-[\alpha]_{no})/ [\alpha]_{no}$	-0.51%	-0.12%	0.09%	0.47%	0.07%	1.65%	1.42%	2.05%	1.41%	1.18%	2.20%	2.62%	1.93%	1.60%	2.21%	1.20%	0.0098
250	α_E	0.268	0.270	0.270	0.269	0.270	0.272	0.273	0.273	0.273	0.274	0.270	0.272	0.272	0.271	0.272	0.271	0.0017
	$[\alpha]_E$	53.572	53.725	53.931	53.841	53.974	54.283	54.649	54.586	54.53	54.892	54.041	54.554	54.567	54.342	54.617	54.274	0.3978
	$C_{\alpha E}$	37.38%	37.54%	37.76%	37.66%	37.81%	38.14%	38.53%	38.46%	38.40%	38.79%	37.88%	38.43%	38.44%	38.20%	38.50%	38.13%	0.0043
	$C_{\beta E}$	62.62%	62.46%	62.24%	62.34%	62.19%	61.86%	61.47%	61.54%	61.60%	61.21%	62.12%	61.57%	61.56%	61.80%	61.50%	61.87%	0.0043
0	α_{no}	0.270	0.270	0.270	0.270	0.271	0.269	0.268	0.270	0.270	0.269	0.269	0.270	0.269	0.269	0.268	0.269	0.0008
	$[\alpha]_{no}$	53.761	54.08	53.881	54.044	54.041	53.931	53.618	54.287	54.107	53.931	53.918	53.976	53.987	54.112	54.014	53.979	0.1568
	$C_{\alpha no}$	37.58%	37.92%	37.71%	37.88%	37.88%	37.76%	37.43%	38.14%	37.95%	37.76%	37.75%	37.81%	37.82%	37.95%	37.85%	37.81%	0.0017
	$C_{\beta no}$	62.42%	62.08%	62.29%	62.12%	62.12%	62.24%	62.57%	61.86%	62.05%	62.24%	62.25%	62.19%	62.18%	62.05%	62.15%	62.19%	0.0017
	$(C_{\alpha E}-C_{\alpha no})/ C_{\alpha no}$	-0.54%	-1.00%	0.14%	-0.57%	-0.19%	1.00%	2.95%	0.84%	1.19%	2.73%	0.35%	1.64%	1.64%	0.65%	1.71%	0.83%	0.0118
	$(C_{\beta E}-C_{\beta no})/ C_{\beta no}$	0.32%	0.61%	-0.09%	0.35%	0.12%	-0.61%	-1.77%	-0.52%	-0.73%	-1.65%	-0.21%	-1.00%	-1.00%	-0.40%	-1.04%	-0.51%	0.0071
	$([\alpha]_E-[\alpha]_{no})/ [\alpha]_{no}$	-0.35%	-0.66%	0.09%	-0.38%	-0.12%	0.65%	1.92%	0.55%	0.78%	1.78%	0.23%	1.07%	1.07%	0.43%	1.12%	0.55%	0.0077

Table S5 The temperature is 25℃

Electric field intensity (V/m)	Parameter	1	2	3	4	5	6	7	8	9	10	11	12	13	14	15	Average	Standard deviation
31.25	α_E	0.275	0.275	0.276	0.275	0.276	0.275	0.275	0.276	0.275	0.274	0.275	0.275	0.274	0.275	0.275	0.275	0.0006
	$[\alpha]_E$	55.045	55.191	55.321	54.38	54.802	55.002	54.946	54.942	55.188	54.526	55.114	55.142	55.384	55.014	55.117	55.008	0.2706
	$C_{\alpha E}$	38.95%	39.11%	39.25%	38.24%	38.69%	38.91%	38.85%	38.84%	39.11%	38.40%	39.03%	39.06%	39.32%	38.92%	39.03%	38.91%	0.0029
	$C_{\beta E}$	61.05%	60.89%	60.75%	61.76%	61.31%	61.09%	61.15%	61.16%	60.89%	61.60%	60.97%	60.94%	60.68%	61.08%	60.97%	61.09%	0.0029
0	α_{no}	0.274	0.273	0.273	0.273	0.272	0.269	0.266	0.269	0.267	0.268	0.269	0.268	0.270	0.269	0.269	0.270	0.0025
	$[\alpha]_{no}$	54.902	54.5	54.38	54.453	54.453	53.638	53.472	53.442	53.382	53.762	53.942	53.577	53.987	53.587	53.789	53.951	0.4736
	$C_{\alpha no}$	38.80%	38.37%	38.24%	38.32%	38.32%	37.45%	37.27%	37.24%	37.17%	37.58%	37.77%	37.38%	37.82%	37.39%	37.61%	37.78%	0.0051
	$C_{\beta no}$	61.20%	61.63%	61.76%	61.68%	61.68%	62.55%	62.73%	62.76%	62.83%	62.42%	62.23%	62.62%	62.18%	62.61%	62.39%	62.22%	0.0051
	$(C_{\alpha E}-C_{\alpha no})/ C_{\alpha no}$	0.40%	1.93%	2.64%	-0.20%	0.98%	3.90%	4.24%	4.32%	5.21%	2.18%	3.33%	4.49%	3.96%	4.09%	3.78%	3.00%	0.0163
	$(C_{\beta E}-C_{\beta no})/ C_{\beta no}$	-0.25%	-1.20%	-1.63%	0.13%	-0.61%	-2.34%	-2.52%	-2.56%	-3.08%	-1.31%	-2.02%	-2.68%	-2.41%	-2.44%	-2.28%	-1.82%	0.0096
	$([\alpha]_E-[\alpha]_{no})/ [\alpha]_{no}$	0.26%	1.27%	1.73%	-0.13%	0.64%	2.54%	2.76%	2.81%	3.38%	1.42%	2.17%	2.92%	2.59%	2.66%	2.47%	1.96%	0.0106
62.5	α_E	0.273	0.270	0.275	0.272	0.273	0.265	0.264	0.264	0.267	0.263	0.280	0.277	0.278	0.281	0.280	0.272	0.0064
	$[\alpha]_E$	54.145	54.267	54.545	54.28	54.663	52.933	52.986	52.71	53.166	52.487	56.013	55.647	55.893	55.72	55.73	54.346	1.2601
	$C_{\alpha E}$	37.99%	38.12%	38.42%	38.14%	38.55%	36.69%	36.75%	36.45%	36.94%	36.21%	39.99%	39.60%	39.86%	39.68%	39.69%	38.21%	0.0135
	$C_{\beta E}$	62.01%	61.88%	61.58%	61.86%	61.45%	63.31%	63.25%	63.55%	63.06%	63.79%	60.01%	60.40%	60.14%	60.32%	60.31%	61.79%	0.0135
0	α_{no}	0.260	0.269	0.259	0.264	0.265	0.261	0.261	0.260	0.261	0.261	0.282	0.285	0.280	0.280	0.279	0.268	0.0097
	$[\alpha]_{no}$	53.644	53.844	54.044	53.06	53.562	52.314	51.206	51.525	51.746	51.566	56.436	56.854	55.88	56.266	56.236	53.879	2.0096
	$C_{\alpha no}$	37.45%	37.67%	37.88%	36.83%	37.37%	36.03%	34.84%	35.18%	35.42%	35.23%	40.45%	40.89%	39.85%	40.26%	40.23%	37.71%	0.0215
	$C_{\beta no}$	62.55%	62.33%	62.12%	63.17%	62.63%	63.97%	65.16%	64.82%	64.58%	64.77%	59.55%	59.11%	60.15%	59.74%	59.77%	62.29%	0.0215
	$(C_{\alpha E}-C_{\alpha no})/ C_{\alpha no}$	1.43%	1.20%	1.42%	3.55%	3.16%	1.84%	5.48%	3.61%	4.30%	2.80%	-1.12%	-3.16%	0.03%	-1.45%	-1.35%	1.33%	0.0246
	$(C_{\beta E}-C_{\beta no})/ C_{\beta no}$	-0.86%	-0.73%	-0.86%	-2.07%	-1.88%	-1.04%	-2.93%	-1.96%	-2.36%	-1.52%	0.76%	2.19%	-0.02%	0.98%	0.91%	-0.80%	0.0146
	$([\alpha]_E-[\alpha]_{no})/ [\alpha]_{no}$	0.93%	0.79%	0.93%	2.30%	2.06%	1.18%	3.48%	2.30%	2.74%	1.79%	-0.75%	-2.12%	0.02%	-0.97%	-0.90%	0.87%	0.0159
125	α_E	0.267	0.270	0.265	0.267	0.267	0.267	0.268	0.268	0.267	0.267	0.267	0.267	0.268	0.268	0.267	0.267	0.0010
	$[\alpha]_E$	53.276	54.257	53.046	53.179	53.143	53.512	53.861	53.369	53.502	53.179	53.861	53.492	53.761	53.898	53.776	53.541	0.3504
	$C_{\alpha E}$	37.06%	38.11%	36.81%	36.95%	36.92%	37.31%	37.69%	37.16%	37.30%	36.95%	37.69%	37.29%	37.58%	37.73%	37.59%	37.34%	0.0038
	$C_{\beta E}$	62.94%	61.89%	63.19%	63.05%	63.08%	62.69%	62.31%	62.84%	62.70%	63.05%	62.31%	62.71%	62.42%	62.27%	62.41%	62.66%	0.0038
0	α_{no}	0.263	0.265	0.265	0.265	0.264	0.264	0.265	0.264	0.266	0.264	0.264	0.264	0.265	0.263	0.263	0.264	0.0009
	$[\alpha]_{no}$	52.707	52.763	52.644	52.983	52.807	52.96	52.863	52.827	52.94	52.6	52.883	52.948	52.77	52.831	52.556	52.805	0.1330
	$C_{\alpha no}$	36.45%	36.51%	36.38%	36.74%	36.56%	36.72%	36.62%	36.58%	36.70%	36.33%	36.64%	36.71%	36.52%	36.58%	36.29%	36.55%	0.0014
	$C_{\beta no}$	63.55%	63.49%	63.62%	63.26%	63.44%	63.28%	63.38%	63.42%	63.30%	63.67%	63.36%	63.29%	63.48%	63.42%	63.71%	63.45%	0.0014
	$(C_{\alpha E}-C_{\alpha no})/ C_{\alpha no}$	1.67%	4.39%	1.18%	0.57%	0.99%	1.61%	2.92%	1.59%	1.64%	1.71%	2.86%	1.59%	2.91%	3.13%	3.60%	2.16%	0.0107
	$(C_{\beta E}-C_{\beta no})/ C_{\beta no}$	-0.96%	-2.52%	-0.68%	-0.33%	-0.57%	-0.93%	-1.69%	-0.92%	-0.95%	-0.97%	-1.65%	-0.92%	-1.67%	-1.80%	-2.05%	-1.24%	0.0062
	$([\alpha]_E-[\alpha]_{no})/ [\alpha]_{no}$	1.08%	2.83%	0.76%	0.37%	0.64%	1.04%	1.89%	1.03%	1.06%	1.10%	1.85%	1.03%	1.88%	2.02%	2.32%	1.39%	0.0069

Continuation of table S5 The temperature is 25℃

Electric field intensity (V/m)	Parameter	1	2	3	4	5	6	7	8	9	10	11	12	13	14	15	Average	Standard deviation
187.5	α_E	0.263	0.264	0.261	0.263	0.262	0.283	0.283	0.282	0.282	0.282	0.283	0.283	0.282	0.281	0.282	0.276	0.0096
	$[\alpha]_E$	52.527	52.724	52.441	52.434	52.271	56.657	56.498	56.03	56.359	56.396	56.342	56.165	56.224	56.031	56.398	55.033	1.8781
	$C_{\alpha E}$	36.26%	36.47%	36.16%	36.16%	35.98%	40.68%	40.51%	40.01%	40.36%	40.40%	40.35%	40.16%	40.22%	40.01%	40.41%	38.94%	0.0201
	$C_{\beta E}$	63.74%	63.53%	63.84%	63.84%	64.02%	59.32%	59.49%	59.99%	59.64%	59.60%	59.65%	59.84%	59.78%	59.99%	59.59%	61.06%	0.0201
0	α_{no}	0.265	0.262	0.261	0.262	0.262	0.280	0.281	0.280	0.281	0.279	0.280	0.279	0.279	0.280	0.280	0.274	0.0086
	$[\alpha]_{no}$	52.737	52.624	52.444	52.534	52.564	55.867	55.774	55.737	55.897	55.677	55.981	55.394	55.761	55.859	55.985	54.722	1.5748
	$C_{\alpha no}$	36.48%	36.36%	36.17%	36.26%	36.30%	39.84%	39.74%	39.70%	39.87%	39.63%	39.96%	39.33%	39.72%	39.83%	39.96%	38.61%	0.0169
	$C_{\beta no}$	63.52%	63.64%	63.83%	63.74%	63.70%	60.16%	60.26%	60.30%	60.13%	60.37%	60.04%	60.67%	60.28%	60.17%	60.04%	61.39%	0.0169
	$(C_{\alpha E}-C_{\alpha no})/ C_{\alpha no}$	-0.62%	0.29%	-0.01%	-0.30%	-0.87%	2.13%	1.95%	0.79%	1.24%	1.94%	0.97%	2.10%	1.25%	0.46%	1.11%	0.86%	0.0099
	$(C_{\beta E}-C_{\beta no})/ C_{\beta no}$	0.35%	-0.17%	0.01%	0.17%	0.49%	-1.41%	-1.29%	-0.52%	-0.82%	-1.28%	-0.64%	-1.36%	-0.82%	-0.31%	-0.74%	-0.54%	0.0063
	$([\alpha]_E-[\alpha]_{no})/ [\alpha]_{no}$	-0.40%	0.19%	-0.01%	-0.19%	-0.56%	1.41%	1.30%	0.53%	0.83%	1.29%	0.64%	1.39%	0.83%	0.31%	0.74%	0.57%	0.0065
250	α_E	0.274	0.279	0.270	0.276	0.271	0.272	0.273	0.271	0.271	0.273	0.272	0.272	0.271	0.272	0.271	0.273	0.0023
	$[\alpha]_E$	54.809	55.887	53.858	55.385	54.051	54.327	54.214	54.097	54.217	54.2	54.317	54.211	54.085	54.369	54.117	54.410	0.5471
	$C_{\alpha E}$	38.70%	39.86%	37.68%	39.32%	37.89%	38.19%	38.06%	37.94%	38.07%	38.05%	38.17%	38.06%	37.93%	38.23%	37.96%	38.27%	0.0059
	$C_{\beta E}$	61.30%	60.14%	62.32%	60.68%	62.11%	61.81%	61.94%	62.06%	61.93%	61.95%	61.83%	61.94%	62.07%	61.77%	62.04%	61.73%	0.0059
0	α_{no}	0.268	0.268	0.267	0.269	0.269	0.271	0.271	0.270	0.270	0.269	0.270	0.271	0.271	0.270	0.270	0.270	0.0012
	$[\alpha]_{no}$	53.495	53.229	53.322	53.701	53.618	53.994	53.715	54.107	54.117	53.848	53.984	53.876	54.008	53.858	53.774	53.776	0.2687
	$C_{\alpha no}$	37.29%	37.01%	37.11%	37.51%	37.43%	37.83%	37.53%	37.95%	37.96%	37.67%	37.82%	37.70%	37.84%	37.68%	37.59%	37.60%	0.0029
	$C_{\beta no}$	62.71%	62.99%	62.89%	62.49%	62.57%	62.17%	62.47%	62.05%	62.04%	62.33%	62.18%	62.30%	62.16%	62.32%	62.41%	62.40%	0.0029
	$(C_{\alpha E}-C_{\alpha no})/ C_{\alpha no}$	3.78%	7.70%	1.55%	4.81%	1.24%	0.94%	1.43%	-0.03%	0.28%	1.00%	0.94%	0.95%	0.22%	1.45%	0.98%	1.81%	0.0207
	$(C_{\beta E}-C_{\beta no})/ C_{\beta no}$	-2.25%	-4.52%	-0.91%	-2.89%	-0.74%	-0.57%	-0.86%	0.02%	-0.17%	-0.61%	-0.57%	-0.58%	-0.13%	-0.88%	-0.59%	-1.09%	0.0122
	$([\alpha]_E-[\alpha]_{no})/ [\alpha]_{no}$	2.46%	4.99%	1.01%	3.14%	0.81%	0.62%	0.93%	-0.02%	0.18%	0.65%	0.62%	0.62%	0.14%	0.95%	0.64%	1.18%	0.0134

Table S6 The temperature is 30℃

Electric field intensity (V/m)	Parameter	1	2	3	4	5	6	7	8	9	10	11	12	13	14	15	Average	Standard deviation
31.25	α_E	0.269	0.268	0.272	0.269	0.272	0.270	0.271	0.270	0.271	0.271	0.270	0.270	0.270	0.271	0.271	0.270	0.0011
	$[\alpha]_E$	54.001	54.200	53.955	54.147	54.101	53.761	53.475	54.417	53.788	54.682	54.002	54.145	54.116	54.324	54.287	54.093	0.2905
	$C_{\alpha E}$	37.84%	38.05%	37.79%	37.99%	37.94%	37.58%	37.27%	38.28%	37.61%	38.57%	37.84%	37.99%	37.96%	38.18%	38.14%	37.94%	0.0031
	$C_{\beta E}$	62.16%	61.95%	62.21%	62.01%	62.06%	62.42%	62.73%	61.72%	62.39%	61.43%	62.16%	62.01%	62.04%	61.82%	61.86%	62.06%	0.0031
0	α_{no}	0.269	0.271	0.270	0.269	0.270	0.269	0.270	0.270	0.270	0.269	0.269	0.270	0.270	0.270	0.270	0.270	0.0006
	$[\alpha]_{no}$	53.828	54.18	53.934	54.042	54.028	54.147	54.007	53.961	53.848	54.21	53.887	54.015	54.174	54.116	54.017	54.026	0.1213
	$C_{\alpha no}$	37.65%	38.03%	37.76%	37.88%	37.86%	37.99%	37.84%	37.79%	37.67%	38.06%	37.71%	37.85%	38.02%	37.96%	37.85%	37.86%	0.0013
	$C_{\beta no}$	62.35%	61.97%	62.24%	62.12%	62.14%	62.01%	62.16%	62.21%	62.33%	61.94%	62.29%	62.15%	61.98%	62.04%	62.15%	62.14%	0.0013
	$(C_{\alpha E}-C_{\alpha no})/ C_{\alpha no}$	0.49%	0.06%	0.06%	0.30%	0.21%	-1.09%	-1.51%	1.29%	-0.17%	1.33%	0.33%	0.37%	-0.16%	0.59%	0.76%	0.19%	0.0075
	$(C_{\beta E}-C_{\beta no})/ C_{\beta no}$	-0.30%	-0.03%	-0.04%	-0.18%	-0.13%	0.67%	0.92%	-0.79%	0.10%	-0.82%	-0.20%	-0.22%	0.10%	-0.36%	-0.47%	-0.12%	0.0046
	$([\alpha]_E-[\alpha]_{no})/ [\alpha]_{no}$	0.32%	0.04%	0.04%	0.19%	0.14%	-0.71%	-0.99%	0.85%	-0.11%	0.87%	0.21%	0.24%	-0.11%	0.38%	0.50%	0.12%	0.0049
62.5	α_E	0.269	0.270	0.271	0.267	0.272	0.267	0.269	0.265	0.266	0.266	0.268	0.267	0.269	0.268	0.268	0.268	0.0019
	$[\alpha]_E$	53.648	53.841	54.413	53.562	54.237	53.163	53.535	53.126	53.402	53.362	53.554	53.532	53.845	53.777	53.604	53.640	0.3503
	$C_{\alpha E}$	37.46%	37.66%	38.28%	37.37%	38.09%	36.94%	37.34%	36.90%	37.19%	37.15%	37.36%	37.33%	37.67%	37.60%	37.41%	37.45%	0.0038
	$C_{\beta E}$	62.54%	62.34%	61.72%	62.63%	61.91%	63.06%	62.66%	63.10%	62.81%	62.85%	62.64%	62.67%	62.33%	62.40%	62.59%	62.55%	0.0038
0	α_{no}	0.269	0.269	0.268	0.269	0.268	0.267	0.266	0.266	0.266	0.265	0.266	0.267	0.266	0.266	0.265	0.267	0.0014
	$[\alpha]_{no}$	53.941	53.685	53.442	53.821	53.602	53.462	53.222	53.182	53.209	52.943	53.014	53.224	53.212	53.117	52.989	53.338	0.3080
	$C_{\alpha no}$	37.77%	37.50%	37.24%	37.64%	37.41%	37.26%	37.00%	36.96%	36.99%	36.70%	36.78%	37.00%	36.99%	36.89%	36.75%	37.13%	0.0033
	$C_{\beta no}$	62.23%	62.50%	62.76%	62.36%	62.59%	62.74%	63.00%	63.04%	63.01%	63.30%	63.22%	63.00%	63.01%	63.11%	63.25%	62.87%	0.0033
	$(C_{\alpha E}-C_{\alpha no})/ C_{\alpha no}$	-0.83%	0.45%	2.79%	-0.74%	1.82%	-0.86%	0.91%	-0.16%	0.56%	1.22%	1.57%	0.89%	1.83%	1.92%	1.79%	0.87%	0.0113
	$(C_{\beta E}-C_{\beta no})/ C_{\beta no}$	0.50%	-0.27%	-1.66%	0.45%	-1.09%	0.51%	-0.53%	0.10%	-0.33%	-0.71%	-0.92%	-0.52%	-1.08%	-1.12%	-1.04%	-0.52%	0.0067
	$([\alpha]_E-[\alpha]_{no})/ [\alpha]_{no}$	-0.54%	0.29%	1.82%	-0.48%	1.18%	-0.56%	0.59%	-0.11%	0.36%	0.79%	1.02%	0.58%	1.19%	1.24%	1.16%	0.57%	0.0074
125	α_E	0.275	0.276	0.274	0.275	0.276	0.271	0.269	0.269	0.270	0.270	0.264	0.263	0.267	0.263	0.264	0.270	0.0048
	$[\alpha]_E$	55.018	55.155	54.716	54.819	55.418	54.42	53.858	53.821	54.12	54.1	52.753	52.504	53.066	52.558	52.714	53.936	1.0055
	$C_{\alpha E}$	38.93%	39.07%	38.60%	38.71%	39.35%	38.29%	37.68%	37.64%	37.96%	37.94%	36.50%	36.23%	36.83%	36.29%	36.46%	37.77%	0.0108
	$C_{\beta E}$	61.07%	60.93%	61.40%	61.29%	60.65%	61.71%	62.32%	62.36%	62.04%	62.06%	63.50%	63.77%	63.17%	63.71%	63.54%	62.23%	0.0108
0	α_{no}	0.271	0.272	0.270	0.269	0.270	0.268	0.266	0.266	0.266	0.267	0.265	0.266	0.265	0.265	0.266	0.267	0.0024
	$[\alpha]_{no}$	53.901	54.267	53.881	53.661	53.724	53.425	53.166	52.963	53.195	53.222	52.963	52.844	52.932	52.887	53.083	53.341	0.4438
	$C_{\alpha no}$	37.73%	38.12%	37.71%	37.47%	37.54%	37.22%	36.94%	36.72%	36.97%	37.00%	36.72%	36.60%	36.69%	36.64%	36.85%	37.13%	0.0048
	$C_{\beta no}$	62.27%	61.88%	62.29%	62.53%	62.46%	62.78%	63.06%	63.28%	63.03%	63.00%	63.28%	63.40%	63.31%	63.36%	63.15%	62.87%	0.0048
	$(C_{\alpha E}-C_{\alpha no})/ C_{\alpha no}$	3.17%	2.50%	2.37%	3.31%	4.84%	2.87%	2.01%	2.50%	2.68%	2.54%	-0.61%	-1.00%	0.39%	-0.96%	-1.07%	1.72%	0.0186
	$(C_{\beta E}-C_{\beta no})/ C_{\beta no}$	-1.92%	-1.54%	-1.44%	-1.98%	-2.91%	-1.70%	-1.18%	-1.45%	-1.57%	-1.49%	0.36%	0.57%	-0.23%	0.56%	0.63%	-1.01%	0.0111
	$([\alpha]_E-[\alpha]_{no})/ [\alpha]_{no}$	2.07%	1.64%	1.55%	2.16%	3.15%	1.86%	1.30%	1.62%	1.74%	1.65%	-0.40%	-0.64%	0.25%	-0.62%	-0.70%	1.12%	0.0121

Continuation of table S6 The temperature is 30℃

Electric field intensity (V/m)	Parameter	1	2	3	4	5	6	7	8	9	10	11	12	13	14	15	Average	Standard deviation
	α_E	0.270	0.269	0.268	0.270	0.267	0.265	0.270	0.268	0.269	0.270	0.268	0.268	0.269	0.270	0.268	0.269	0.0014
187.5	$[\alpha]_E$	54.127	53.685	53.482	53.668	53.379	52.724	53.795	53.678	54.13	53.921	53.586	53.444	53.872	54.011	53.654	53.677	0.3514
	$C_{\alpha E}$	37.97%	37.50%	37.28%	37.48%	37.17%	36.47%	37.62%	37.49%	37.97%	37.75%	37.39%	37.24%	37.70%	37.85%	37.46%	37.49%	0.0038
	$C_{\beta E}$	62.03%	62.50%	62.72%	62.52%	62.83%	63.53%	62.38%	62.51%	62.03%	62.25%	62.61%	62.76%	62.30%	62.15%	62.54%	62.51%	0.0038
	α_{no}	0.269	0.268	0.268	0.267	0.267	0.251	0.259	0.259	0.257	0.258	0.266	0.264	0.266	0.265	0.266	0.263	0.0052
0	$[\alpha]_{no}$	53.592	53.821	53.661	53.502	53.322	50.305	51.506	51.579	51.449	51.546	53.114	52.838	53.214	52.957	53.114	52.635	1.0673
	$C_{\alpha no}$	37.40%	37.64%	37.47%	37.30%	37.11%	33.87%	35.16%	35.24%	35.10%	35.20%	36.89%	36.59%	36.99%	36.72%	36.89%	36.37%	0.0114
	$C_{\beta no}$	62.60%	62.36%	62.53%	62.70%	62.89%	66.13%	64.84%	64.76%	64.90%	64.80%	63.11%	63.41%	63.01%	63.28%	63.11%	63.63%	0.0114
	$(C_{\alpha E}-C_{\alpha no})/ C_{\alpha no}$	1.53%	-0.39%	-0.51%	0.48%	0.16%	7.65%	6.98%	6.38%	8.19%	7.23%	1.37%	1.78%	1.91%	3.08%	1.57%	3.07%	0.0317
	$(C_{\beta E}-C_{\beta no})/ C_{\beta no}$	-0.92%	0.23%	0.31%	-0.28%	-0.10%	-3.92%	-3.78%	-3.47%	-4.43%	-3.93%	-0.80%	-1.02%	-1.12%	-1.79%	-0.92%	-1.76%	0.0169
	$([\alpha]_E-[\alpha]_{no})/ [\alpha]_{no}$	1.00%	-0.25%	-0.33%	0.31%	0.11%	4.81%	4.44%	4.07%	5.21%	4.61%	0.89%	1.15%	1.24%	1.99%	1.02%	1.98%	0.0201
	α_E	0.267	0.271	0.273	0.272	0.266	0.269	0.274	0.270	0.270	0.267	0.269	0.271	0.267	0.267	0.267	0.269	0.0025
250	$[\alpha]_E$	53.106	54.064	54.433	54.383	53.173	53.941	54.736	53.791	54.184	53.489	53.598	54.137	53.588	53.342	53.412	53.825	0.4910
	$C_{\alpha E}$	36.88%	37.90%	38.30%	38.25%	36.95%	37.77%	38.62%	37.61%	38.03%	37.29%	37.40%	37.98%	37.39%	37.13%	37.20%	37.65%	0.0053
	$C_{\beta E}$	63.12%	62.10%	61.70%	61.75%	63.05%	62.23%	61.38%	62.39%	61.97%	62.71%	62.60%	62.02%	62.61%	62.87%	62.80%	62.35%	0.0053
	α_{no}	0.264	0.264	0.264	0.264	0.263	0.269	0.266	0.269	0.267	0.270	0.263	0.262	0.263	0.262	0.261	0.265	0.0028
0	$[\alpha]_{no}$	52.724	52.474	52.318	52.381	52.315	53.552	53.07	53.705	53.116	54.014	52.398	52.387	52.454	52.444	52.225	52.772	0.5784
	$C_{\alpha no}$	36.47%	36.20%	36.03%	36.10%	36.03%	37.35%	36.84%	37.52%	36.89%	37.85%	36.12%	36.11%	36.18%	36.17%	35.93%	36.52%	0.0062
	$C_{\beta no}$	63.53%	63.80%	63.97%	63.90%	63.97%	62.65%	63.16%	62.48%	63.11%	62.15%	63.88%	63.89%	63.82%	63.83%	64.07%	63.48%	0.0062
	$(C_{\alpha E}-C_{\alpha no})/ C_{\alpha no}$	1.12%	4.71%	6.29%	5.94%	2.55%	1.12%	4.85%	0.25%	3.10%	-1.49%	3.56%	5.19%	3.36%	2.66%	3.54%	3.09%	0.0218
	$(C_{\beta E}-C_{\beta no})/ C_{\beta no}$	-0.64%	-2.67%	-3.54%	-3.36%	-1.44%	-0.67%	-2.83%	-0.15%	-1.81%	0.91%	-2.01%	-2.94%	-1.90%	-1.51%	-1.99%	-1.78%	0.0124
	$([\alpha]_E-[\alpha]_{no})/ [\alpha]_{no}$	0.72%	3.03%	4.04%	3.82%	1.64%	0.73%	3.14%	0.16%	2.01%	-0.97%	2.29%	3.34%	2.16%	1.71%	2.27%	2.00%	0.0140
